# Efficacy of extracorporeal shock wave therapy for knee tendinopathies and other soft tissue disorders: a meta-analysis of randomized controlled trials

**DOI:** 10.1186/s12891-018-2204-6

**Published:** 2018-08-02

**Authors:** Chun-De Liao, Guo-Min Xie, Jau-Yih Tsauo, Hung-Chou Chen, Tsan-Hon Liou

**Affiliations:** 1 School and Graduate Institute of Physical Therapy, College of Medicine, National Taiwan University, Taipei, Taiwan; 2 Department of Physical Medicine and Rehabilitation, Shuang Ho Hospital, Taipei Medical University, Taipei, Taiwan; 3 Department of Neurology, Ningbo Medical Center Lihuili Eastern Hospital, Taipei Medical University, Zhejiang, China; 4 Center for Evidence-Based Health Care, Shuang Ho Hospital, Taipei Medical University, Taipei, Taiwan; 5 Graduate Institute of Injury Prevention and Control, Taipei Medical University, Taipei, Taiwan; 6 Department of Physical Medicine and Rehabilitation, School of Medicine, College of Medicine, Taipei Medical University, No. 250 Wu-Hsing Street, Taipei, Taiwan

**Keywords:** Extracorporeal shock wave therapy, Knee, Musculoskeletal disorders, Physical therapy

## Abstract

**Background:**

Extracorporeal shock-wave therapy (ESWT), which can be divided into radial shock-wave therapy (RaSWT) and focused shock-wave therapy (FoSWT), has been widely used in clinical practice for managing orthopedic conditions. The aim of this study was to determine the clinical efficacy of ESWT for knee soft tissue disorders (KSTDs) and compare the efficacy of different shock-wave types, energy levels, and intervention durations.

**Methods:**

We performed a comprehensive search of online databases and search engines without restrictions on the publication year or language. We selected randomized controlled trials (RCTs) reporting the efficacy of ESWT for KSTDs and included them in a meta-analysis and risk of bias assessment. The pooled effect sizes of ESWT were estimated by computing odds ratios (ORs) with 95% confidence intervals (CIs) for the treatment success rate (TSR) and standardized mean differences (SMDs) with 95% CIs for pain reduction (i.e., the difference in pain relief, which was the change in pain from baseline to the end of RCTs between treatment and control groups) and for restoration of knee range of motion (ROM).

**Results:**

We included 19 RCTs, all of which were of high or medium methodological quality and had a Physiotherapy Evidence Database score of ≥5/10. In general, ESWT had overall significant effects on the TSR (OR: 3.36, 95% CI: 1.84–6.12, *P* < 0.0001), pain reduction (SMD: − 1.49, 95% CI: − 2.11 to − 0.87, *P* < 0.00001), and ROM restoration (SMD: 1.76, 95% CI: 1.43–2.09, *P* < 0.00001). Subgroup analyses revealed that FoSWT and RaSWT applied for a long period (≥1 month) had significant effects on pain reduction, with the corresponding SMDs being − 3.13 (95% CI: − 5.70 to − 0.56; *P* = 0.02) and − 1.80 (95% CI: − 2.52 to − 1.08; *P* < 0.00001), respectively. Low-energy FoSWT may have greater efficacy for the TSR than high-energy FoSWT, whereas the inverse result was observed for RaSWT.

**Conclusions:**

The ESWT exerts an overall effect on the TSR, pain reduction, and ROM restoration in patients with KSTDs. Shock-wave types and application levels have different contributions to treatment efficacy for KSTDs, which must be investigated further for optimizing these treatments in clinical practice.

**Electronic supplementary material:**

The online version of this article (10.1186/s12891-018-2204-6) contains supplementary material, which is available to authorized users.

## Background

Knee soft tissue disorders (KSTDs) are common problems that develop from sports-induced tendon and ligament injuries in athletes [1], and they originate from overuse conditions or traumatic injuries in nonathletes [2–4]. Overall, knee injuries account for up to 35% of common overuse injuries in sports teams [5]. The most common practical problem caused by knee injury is the pain-induced limitation in sports and related activities, particularly walking or running [2, 6]; this problem further exerts negative effects on not only sports participation but also quality of life [7, 8].

Over the past three decades, extracorporeal shock wave therapy (ESWT) has been widely used in clinical practice for managing musculoskeletal disorders, most of which are tendinopathies and enthesopathies [9–13]. Because of its efficacy in exerting analgesic effects and promoting soft tissue remodeling and repair, ESWT has also been successfully used for treating many other soft tissue disorders that occur after sports injuries and traumatic accidents, such as muscular disorders [14, 15], posttraumatic knee stiffness [16, 17], and ligament injuries [18–21], as well as ligament desmitis in animals [22–24]. In addition, for orthopedic conditions, ESWT serves as a noninvasive alternative to conservative treatment (i.e., steroid injections) or surgery [25, 26]. ESWT provides a mechanical stimulus that is conducted by pulse acoustic waves, and through mechanotransduction, this stimulus is converted into a series of biochemical signals within the targeted tissues, enhancing tissue regeneration [9, 13, 27]. Consequently, the production of proteins, nitric oxide, and specific growth factors causes responses leading to increased neoangiogenesis, tenocyte and fibroblast proliferation, and collagen synthesis, further enhancing tissue catabolism, healing, and remodeling [28–33]. Acoustic cavitation formed in the negative (tensile) phase of the shock wave is the second effect of ESWT; this effect also promotes tissue regeneration by increasing cellular membrane permeability, and it efficiently breaks down calcification deposits (i.e., calculi disintegration) in soft tissues [9, 13, 34]. The aforementioned cascades of biological events support that ESWT can be employed to reduce pain, increase blood flow in ischemic tissues, soften calcified tissues, treat tissue fibrosis, and release adhesions, as well as relieve posttraumatic knee stiffness, thereby improving physical function and performance in sports activities.

On the basis of the delivery pathway for the propagation of acoustic energy through biological tissue, shock wave therapy can be divided into two types: focused shock wave therapy (FoSWT) and radial shock wave therapy (RaSWT) [11, 34, 35]. The differences in the therapeutic effects of FoSWT and RaSWT have been discussed [11, 36–39], and each therapy should be considered an independent modality derived from multiple techniques that generate shock wave pulses [11, 37, 38]. However, it remains unclear whether any difference exists in the therapeutic effects of FoSWT and RaSWT on KSTDs. The intensity at the focal point of the shock wave, which is measured as energy flux density (EFD; mJ/mm^2^) per impulse, may influence the therapeutic effects of ESWT [34, 36]. In clinical practice, the EFD levels of ESWT range from 0.001 to 0.5 mJ/mm^2^ [36, 37, 40, 41]. Administering ESWT repeatedly and at a very high dosage may increase the risk of treatment failure [42] and increase the onset of adverse events [43, 44]. Thus, it is important to enhance the efficiency of ESWT by determining the differences in the efficacy of various ESWT application levels. The overall pooled effects of different shock-wave types and dosage levels on KSTDs should be further investigated.

Several studies have investigated the efficacy of ESWT for lower limb musculoskeletal conditions or knee tendinopathy through systemic reviews or meta-analyses [45–47]. Nevertheless, two of such studies have selected articles published in a specific language [46, 47]. In addition, other than patellar tendinopathy, most KSTDs have not been included in previous meta-analyses, such as pes anserine tendinopathy [48], fabella syndrome [49, 50], popliteal cyamella [51], iliotibial band friction syndrome [52], infrapatellar fat pad syndrome [53], and posttraumatic tendon and ligament stiffness, which contribute to joint contracture [16, 17]. Restrictions on language in the study inclusion criteria may result in a high risk of bias (i.e., language bias) in research areas such as alternative treatment (e.g., ESWT serves as an alternative to conservative medicine for musculoskeletal conditions) [54]. The aim of the current systematic review and meta-analysis was to determine the efficacy of ESWT in reducing pain and improving functional outcomes in patients with KSTDs at immediate (≤1 month), short-term (> 1 month, ≤3 months), medium-term (> 3 month, ≤6 months), and long-term (> 6 months) follow-up (FU). We also performed subgroup analyses to compare the efficacy of ESWT in reducing pain and improving functional outcomes between different shock-wave types, energy levels (i.e., high and low energy), intervention periods [i.e., short (< 1 month) and long (≥1 month)], control group types (i.e., placebo, noninvasive comparison, and invasive comparison), treated populations (i.e., athletes and nonathletes), disease types (i.e., tendinopathy and other KSTDs), and cointervention designs (i.e., monotherapy and cointervention).

## Methods

### Design

This study was conducted in accordance with the Preferred Reporting Items for Systematic Reviews and Meta-Analysis guidelines [55]. A comprehensive search of online databases and search engines was performed up to June 2018. Original research articles on the clinical efficacy of ESWT for KSDTs were aggregated and coded. To minimize publication and language biases, no limitation was imposed on the publication year or language. Primary sources were MEDLINE, PubMed, the Excerpta Medica dataBASE, the Cochrane Library, the Physiotherapy Evidence Database (PEDro), the China Knowledge Resource Integrated Database, and Google Scholar. Secondary sources were papers cited in the articles retrieved from the aforementioned sources and articles published in journals that were not available in the aforementioned databases. The search was restricted to published or in-press articles reporting human studies. If English titles were not provided in non-English articles, they were translated to English by using translation software (Ginger Software, Inc.). Two researchers (CDL and HCC) independently searched for articles, screened studies, and extracted data in a blinded manner. Any disagreements between the researchers were resolved through consensus, with other research team members (JYT and GMX) acting as arbiters.

### Search strategy

We used the following keywords in the Excerpta Medica dataBASE to identify articles reporting studies applying shock wave therapy for KSTDs and associated conditions: [“shock wave therapy” OR “extracorporeal shock wave therapy”] AND [(“knee soft tissue disorder” OR “knee musculoskeletal disorder” OR “patella/patellar/patellofemoral”) OR (“tendinitis/tendinopathy/peritendinopathy” OR “ligament injury/desmitis” OR “apicitis” OR “apophysitis” OR “enthesopathy” OR “plica” OR “tenosynovitis” OR “synovitis” OR “bursitis” OR “iliotibial band friction syndrome” OR “pes anserine tendinopathy” OR “fabella syndrome” OR “popliteal cyamella” OR “Osgood–Schlatter disease” OR “Jumper’s knee”)] AND [“Randomized controlled trial” OR “Randomization”]. The detailed search formulas used for each database are presented in Additional file 1.

### Study selection

The trial inclusion criteria were (1) randomized controlled trials (RCTs); (2) RCTs in which controls received a placebo through sham shock wave application or underwent noninvasive/invasive treatment (e.g., exercise, injections, or surgery); (3) RCTs involving KSTDs including tendinopathy and other noncartilage soft tissue disorders; (4) trials in which the primary outcomes included pain that was measured using a quantifiable scale (e.g., a visual analog scale [VAS]) and the successful treatment rate that was measured using a ranking scale (e.g., the Roles and Maudsley score [56] or Likert-type scale [57, 58]); (5) trials in which the secondary outcomes included physical function and disability that were assessed using questionnaires for patient-reported outcomes (e.g., the Victorian Institute of Sport Assessment-Patella questionnaire [59]) or measured using performance-based testing (e.g., the vertical jump test); and (6) trials containing the following application parameters: wave characteristics, EFD, number of shock impulses, number and duration of treatment sessions, and frequency of treatment. Trials reporting one primary or secondary outcome were included if they also fulfilled other inclusion criteria. If more than one primary or secondary outcome measure was reported for pain or function, respectively, we extracted data for the outcomes of pain (e.g., the VAS) and function (e.g., assessment for activities of daily living), which are considered to be of the greatest importance in patients and to be disease specific [60].

The trial exclusion criteria were (1) animal trials; (2) trials with a non-RCT design such as a case report, case series, or prospective trial without a comparison group; and (3) trials using ESWT to treat knee cartilage disorders such as chondromalacia, meniscus injury, and degenerative osteoarthritis.

### Data extraction

We developed and refined a data extraction sheet for the included trials [37]. Study characteristics, namely the author name, publication year, study design, participants (i.e., sample size, age, sex, and training status), disease type, symptom onset duration, study group interventions and comparison (including cointerventions), FU duration, outcome measures (including assessment tools), and ESWT application parameters, were extracted according to the standardized data extraction sheet [61]. Information on the side effects of ESWT, loss to FU, author conflict of interest disclosures, and funding sources in each trial was also extracted to assess agenda bias and other potential biases [62]. For all included trials, we also confirmed whether the results of each employed outcome measure which was described in the Methods section being fully reported in the Results section to assess bias that may result from selective outcome reporting [62]. One researcher (CDL) extracted the relevant data from the included trials, and another researcher (HCC) reviewed the extracted data. The reviewers contacted the study authors to confirm any necessary information. Any disagreement between the two researchers was resolved through consensus. A third researcher (THL) was consulted if the disagreement persisted.

### Outcome measures

The primary outcomes—pain intensity and the successful treatment rate—were calculated as standardized mean differences (SMDs) and odds ratios (ORs) relative to the placebo or comparison control, respectively. Secondary outcomes—patient-reported and performance-based outcome measures—were also calculated as SMDs relative to the placebo or active control.

### Assessment of methodological quality

The PEDro classification scale was used to assess the risk of bias of the included RCTs [63, 64]. The methodological quality of all included trials was independently assessed by two researchers (CDL and HCC) through the PEDro classification scale. Any disagreement between the two researchers was resolved through consensus. A third researcher (THL) was consulted if the disagreement could not be resolved.

The PEDro classification scale is a valid measure of the methodological quality of clinical RCTs [63], as recommended for nonpharmacological studies [65]; all 10 item scores are summed to yield a total score ranging from 0 to 10 points, where a summary score ≥ 6 points typically defines adequate trial quality [66]. On the basis of the PEDro score, the methodological quality of each included RCT was rated as high (≥7/10), medium (4–6/10), or low (≤3/10) [67].

### Assessment of risk of bias

The same two researchers (CDL and HCC) independently assessed the risk of bias in the included studies by using the Cochrane risk of bias tool [68, 69]. Any difference of opinion was resolved during a consensus meeting; if the difference persisted, a third reviewer (THL) became involved. The following seven bias domains (11 judgement items) related to bias in estimates of intervention effects were assessed [61]: selection bias (i.e., random sequence generation, allocation concealment, and similarity at baseline), performance bias (i.e., blinding of participants and personnel, blinding of therapists or care providers, and avoidance of cointerventions or similar), detection bias (i.e., blinding and timing of outcome assessment); attrition bias (i.e., incomplete outcome data), reporting bias (i.e., selective reporting), agenda bias (i.e., author conflict of interest disclosures), and other sources of potential bias (e.g., unvalidated outcome measures). According to its quality, each included trial was classified to have low, high, or unclear risk of bias [69].

We also examined adverse events, when reported; however, they were not specified a priori. The FU duration was assessed and defined as immediate (≤1 month), short term (> 1 month, ≤3 months), medium term (> 3 months, ≤6 months), and long term (> 6 months).

### Statistical analysis

We computed the effect sizes for the primary and secondary outcome measures in each trial by following the Cochrane Handbook for Systematic Reviews [69]. In each trial, the treatment effect of ESWT (i.e., the effect size) on the primary outcome (i.e., pain score) was estimated based on the changes in the score at each FU time point relative to the baseline score [i.e., difference between the mean scores at pretreatment and FU time point], as well as standard deviations (SDs) in each group. If the exact variance of paired differences was not reported, it was imputed by assuming a correlation coefficient of 0.8 between the baseline and FU pain scores [70, 71]. If data were reported as median (range), they were recalculated algebraically from the trial data for imputing the sample mean and SD [72]. In addition, the pooled effect size of ESWT was estimated by calculating the weighted SMD along with 95% CIs by using the inverse variance-weighted method. Using the methodology of a previous study [67], we categorized the magnitude of the SMD in accordance with the following version of Cohen’s criteria [73], which was proposed by Hopkins [74]: trivial (*d* < 0.20), small (0.20 ≤ *d* < 0.60), medium (0.60 ≤ *d* < 1.20), and large (*d* ≥ 1.20). The OR along with the corresponding 95% CI was estimated for dichotomous outcomes (i.e., successful treatment rate). For the secondary outcomes of physical mobility and disability, the effect size was calculated as the SMD, thus constituting a combined outcome measure without units.

Statistical heterogeneity was assessed using the *I*^2^ statistic, and a result of χ^2^ > 50% and *P* < 0.05 was defined as evidence of significant heterogeneity across trials [75]. Fixed- or random-effects models were used depending on the absence or presence of significant heterogeneity (*P* > 0.05 and *P* < 0.05), respectively.

Subgroup analyses were performed according to the shock-wave type (i.e., FoSWT and RaSWT), energy level (i.e., high and low energy), intervention period [i.e., short (< 1 month) and long (≥1 month)], (i.e., placebo, noninvasive comparison, and invasive comparison), treated populations (i.e., athletes and nonathletes), disease type (i.e., tendinopathy and other KSTDs), and cointervention design (i.e., monotherapy and cointervention) in the included trials. We used a cutoff EFD value of 0.2 mJ/mm^2^ for high and low energy [40], and an EFD range with the upper limit of 0.2 mJ/mm^2^ or higher was also considered as a high energy level.

Using SPSS statistical software (Version 17.0; IBM, Armonk, NY, USA), we investigated potential publication bias through the visual inspection of a funnel plot [76] and Egger’s regression asymmetry test [77]. *P* < 0.05 was considered statistically significant. All analyses were conducted using RevMan 5.3 (The Nordic Cochrane Centre, Copenhagen, Denmark).

We graded the levels of evidence (LoE) for each outcome of interest according to the guideline of evidence synthesis [78] derived from the criteria of van Tulder [79] (Table 1).

**Table 1:** Guidelines of evidence synthesis^a^

Level of evidence	Criteria of judgement
Strong	Provided by consistent^b^, statistically significant pooled results in SMD or OR derived from multiple RCTs, including at least two high-quality RCTs^c^
Moderate	Provided by statistically significant results in one high-quality RCT^c^ ***or*** Provided by inconsistent^b^, statistically significant pooled results in SMD or OR derived from multiple RCTs, including at least one high-quality RCT^c^ ***or*** Provided by consistent^b^, statistically significant pooled results in SMD or OR derived from multiple medium-quality RCTs^c^.
Limited	Provided by statistically significant results in one medium-quality RCT^c^ ***or*** Provided by inconsistent^b^, statistically significant pooled results in SMD or OR derived from multiple RCTs, including at least one medium-quality RCT^c^ ***or*** Provided by consistent^b^, statistically significant pooled results in SMD or OR derived from multiple low-quality RCTs^c^
Very limited	Provided by statistically significant results in one low-quality RCT^c^ ***or*** Provided by inconsistent^b^, statistically significant pooled results in SMD or OR derived from multiple low-quality RCTs^c^
Conflicting	Provided by inconsistent^b^, statistically non-significant results in SMD or OR derived from multiple RCTs regardless of quality

*RCT* randomized controlled trial, *SMD* standard mean difference, *OR* odds ratio

^a^Established in accordance with the “Best-evidence synthesis” which was adapted by Dorrestijn et al. [78] from the van Tulder’s criteria [79]

^b^Pooled results are considered consistent if no statistically significant heterogeneity (*I*^2^, *P* > 0.05) been identified and those are considered inconsistent if statistically significant *I*^2^ (*P <* 0.05) been identified

^c^Methodological quality of a study is rated based on PEDro score as high (≥7/10), medium (4–6/10), and low (≤3/10)

## Results

### Trial selection process

**Fig. 1:**
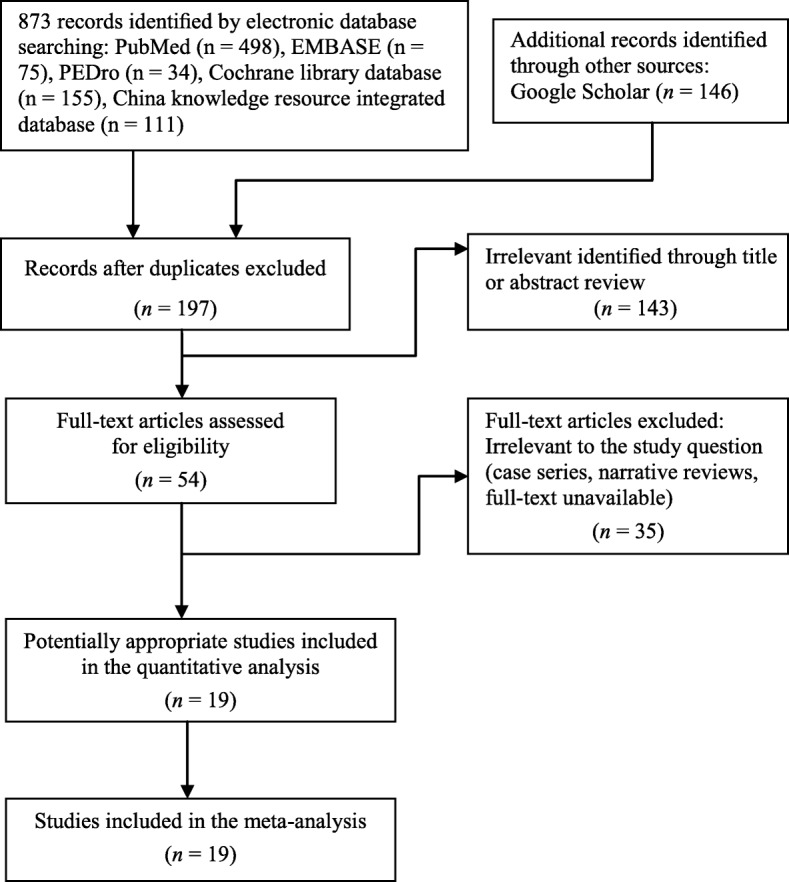
PRISMA flowchart for review and selection of studies

Figure 1 presents a flowchart of the selection process. The final sample for meta-analysis comprised 19 RCTs [16, 17, 48, 51–53, 80–92], totally including 1189 patients [mean (SD) age: 34.7 (9.4) years]. Of all patients, 562 received ESWT and 627 received a placebo or other comparative treatments.

### Study characteristics

Table 2 summarizes the demographic data and study characteristics of the included RCTs. All patients in the included RCTs had experienced symptoms for 3 months or longer, except for those in one RCT, except for those in one RCT, who experienced traumatic knee synovitis for 2 months [92]. ESWT was used to treat orthopedic conditions including patellar tendinopathy (eight RCTs) [81–85, 87, 88, 90], pes anserine tendinopathy (two RCTs) [48, 80], anterior cruciate ligament (ACL) injury (two RCTs) [86, 89], traumatic knee synovitis (one RCT) [92], Osgood–Schlatter disease (one RCT) [91], iliotibial band syndrome (one RCT) [52], and infrapatellar fat pad injury (one RCT) [53]. In addition, it was used to treat posttraumatic knee stiffness (two RCTs) [16, 17] and popliteal cyamella (one RCT), which represents gastrocnemius tendinopathy [51].

**Table 2:** Summary of included study characteristics

Study author (year) [reference]	Groups	Age (years) Mean (SD)	Sex F/M	*N*	Design	Diagnosis	Involved side Unilateral/bilateral	Athlete/nonathlete	Duration of symptoms (months) Mean (SD, range)	Cointervention	Follow up time point	Outcome results	Fund or grant^§^	MQ score^*^
Chen (2014) [51]	EG: ESWT + MSE	63.0 (7.4)^‡^	102/18^‡^	30	RCT, DB	Popliteal cyamella	NR	Nonathlete	10–144^‡^	None	Baseline	VAS^b,c^; ROM^b,c^	Funded	7/10
	CG 1: USD + MSE			30							Posttest: ≤1, 6 months	Lequesne’s index^b,c^		
	CG 2: MSE			30										
	CG 3: Non-ESWT^†^			30										
Geng (2017) [90]	EG: ESWT + APT	35.9 (10.2)^‡^	19/41^‡^	30	RCT	CPT	NR	Nonathlete	4.7 (2.8) ^‡^	None	Baseline	VAS^a,b,c^; PTT^a,b,c^	Funded	6/10
	CG: CT			30							Posttest: 1 month	4-point Likert scale^c^		
Guan (2015) [80]	EG: ESWT	45.5 (20–80)^‡^	91/55^‡^	73	RCT	PAT	128/18^‡^	Nonathlete	12.5 (6–36)^‡^	None	Baseline	VAS^a,b,c^	NR	6/10
	CG: CT			73							Posttest: 6 months			
Huang (2017) [88]	EG: ESWT	22.0 (3.0)	0/31	31	RCT	CPT	28/3	Athlete	12.0 (6–24)	None	Baseline	VAS^a,b,c^; VISA-P^a,b,c^	Funded	6/10
	CG: CT	21.0 (3.0)	0/30	30			29/1		11.0 (6–20)		Posttest: 1, 3, 12 months	4-point Likert scale^c^		
Jiang (2016) [81]	EG: ESWT	35.7 (9.1)	24/16	40	RCT	CPT	40/0	Nonathlete	4.2 (3.9)	PT	Baseline	VAS^a,b,c^; PI^b,c^	NR	6/10
	CG: Non-ESWT^†^	34.4 (10.7)	21/15	36			36/0		4.7 (4.4)	Pain medication	Posttest: ≤1, 2 weeks	KOS-ADLS^a,b,c^		
Khosrawi (2017) [48]	EG: ESWT	49.4 (7.8)	16/4	20	RCT, SB	PAT	NR	Nonathlete	> 3 months^‡^	STE	Baseline	VAS^a,b,c^; MPQ^a,b,c^	Funded	8/10
	CG: Sham ESWT	50.2 (8.1)	15/5	20						Pain medication	Posttest: ≤1, 2 months			
Liu (2016) [82]	EG: ESWT	22.1 (1.5)	22/28	50	RCT, SB	CPT	50/0	Athlete	4.9 (1.3, 3–6)	APT	Baseline	VAS^a,b,c^; VISA-P^a,b,c^	Funded	6/10
	CG: Iontophoresis	22.2 (1.3)	23/27	50			50/0		5.0 (1.1, 3–6)	Massage	Posttest: ≤1, 3, 6, 12 months	4-point RMS^c^		
Taunton (2003) [83]	EG: ESWT	23–52^‡^	5/5	10	RCT, SB	CPT	NR	Athlete	> 3 months ^‡^	None	Baseline	VISA-P^a,b,c^	Funded	5/10
	CG: Sham ESWT		5/5	10							Posttest: ≤1, 3 months	Vertical jump test^b,c^		
Thijs (2017) [84]	EG: ESWT	30.5 (8.0)	8/14	22	RCT, DB	CPT	NR	Nonathlete	16.3 (18.2, 3–78)	ET exercise	Baseline	VAS^a,b^; VISA-P^a,b^	NR	9/10
	CG: Sham ESWT	27.3 (5.2)	6/24	30					24.9 (31.6, 3–125)		Posttest: ≤1, 3, 6 months	6-point Likert scale		
Vetrano (2013) [85]	EG: ESWT	26.8 (8.5)	6/17	23	RCT, SB	CPT	23/0	Athlete	17.6 (20.2)	STE	Baseline	VAS^a,b,c^; VISA-P^a,b,c^	NR	7/10
	CG: PRP	26.9 (9.1)	3/20	23			23/0		18.9 (19.1)		Posttest: 2, 6, 12 months	MBS^a,b,c^		
Wang (2014) [86]	EG: ESWT	28.3 (7.4)	5/21	26	RCT, SB	ACL reconstruction	26/0	Nonathlete	21.4 (22.5, 1–72)	PT	Baseline	LFS^a,b,c^	Funded	8/10
	CG: Non-ESWT^†^	27.7 (7.7)	6/21	27			27/0		15.4 (21.9, 1–84)		Posttest: 12, 24 months	IKDC score^a,b^		
Weckström (2016) [52]	EG: ESWT	23.7 (2.0)	6/14	11	RCT	ITBS	NR	Nonathlete	60.4 (53.7)	MSE	Baseline	11-point NRS^b^;	NR	7/10
	CG: CT	24.2 (2.2)	7/13	13					42.3 (65.1)	STE	Posttest: 1, 2, 12 months	Treadmill test		
Wu (2009) [91]	EG: ESWT	15.9 (11–19)	9/21	30	RCT	OSD	NR	Athlete	3–36	None	Baseline	VAS^a,b,c^; MPQ^a,b,c^	NR	6/10
	CG: USD	16.5 (14.–19)	7/23	30					3–36		Posttest: 0, 3 months	3-point Likert scale^c^		
Wu (2016) [89]	EG: ESWT	26.0 (19–38)^‡^	7/55^‡^	31	RCT	ACL injury	31/0	Nonathlete	3.8 (1–12)^‡^	PT	Baseline	VAS^a,b,c^	NR	6/10
	CG: Non-ESWT^†^			31			31/0				Posttest: ≤1 month	4-point Likert scale^c^		
Yang (2007) [16]	EG: ESWT	34.0 (7.4)	6/22	28	RCT	PTKS	28/0	Nonathlete	6.0 (5.3)	MSE	Baseline	VAS^a,b,c^; ROM^a,b,c^	NR	5/10
	CG: CPM	33.0 (8.4)	9/20	29			29/0		6.0 (3.3)		Posttest: ≤1 month	4-point Likert scale^c^		
Zhang (2016) [92]	EG: ESWT	48.0 (4.6)	10/8	18	RCT, SB	Traumatic synovitis	18/0	Nonathlete	1–2	APT; MSE	Baseline	VAS^a,b,c^; ROM^a,b,c^;	Funded	6/10
	CG: Non-ESWT^†^	50.0 (5.8)	11/7	18			18/0		1–2		Posttest: 2, 4, 6 weeks	Swelling^a,b,c^; LFS^a,b,c^ 4-point Likert scale		
Zhang (2017) [17]	EG: ESWT	34.8 (5.6)	7/21	28	RCT	PTKS	28/0	Nonathlete	4.7 (2.3)	PT	Baseline	VAS^a,b,c^; ROM^a,b,c^;	NR	6/10
	CG: Non-ESWT^†^	35.5 (4.9)	9/17	26			26/0		4.3 (2.6)		Posttest: 0 month	HSS^a,b,c^; 4-point Likert scale		
Zhou (2015) [53]	EG: ESWT	25.0 (18–30)^‡^	30/30^‡^	30	RCT	IPFP injury	50/10	Athlete	24 (1–48)^‡^	None	Baseline	VAS^a,b,c^;	NR	6/10
	CG: APT			30							Posttest: ≤1 month	4-point Likert scale^c^		
Zwerver (2011) [87]	EG: ESWT	24.2 (5.2)	11/20	31	RCT, DB	CPT	18/13	Athlete	7.3 (3.6)	Sports participation	Baseline	VAS ^a,b^; VISA-P ^a,b^;	Funded	9/10
	CG: Sham ESWT	25.7 (4.5)	10/21	31			13/18		8.1 (3.8)	Medical treatment	Posttest: ≤1, 3, 6 months	Knee-loading pain test		

^*^Assessed using the 10-point PEDro classification scale

^†^No application of shock wave treatment

^‡^Value of total sample

^§^Details of the funding information of the studies are presented in Additional file 10: Table S3

^a^Significant improvements in the control group compared with baseline (*P* < 0.05)

^b^Significant improvements in the experimental group compared with baseline (*P* < 0.05)

^c^Significant between-group difference for ESWT compared with control (*P* < 0.05)

*MQ* methodological quality, *EG* experimental group, *CG* control group, *ESWT* extracorporeal shock wave therapy, *RCT* randomized controlled trial, *QRCT* Quasi-randomized controlled trial, *DB* double blind, *VAS* visual analog scale, *NR* not reported, *ET* eccentric training, *VISA-P* Victorian Institute of Sport Assessment-Patella, *PRP* platelet-rich plasma, *PTKS* posttraumatic knee stiffness, *USD* ultrasound diathermy, *MSE* muscular strengthening exercise, *APT* acupuncture therapy, *LPNIR-LI* linear polarized near-infrared light irradiation, *CT* conservative treatment, *STE* stretching exercise, *ITBS* iliotibial band syndrome, *OSD* Osgood–Schlatter disease, *CPT* chronic patellar tendinopathy, *PTT* patellar tendon thickness, *PT* physiotherapy, *PAT* pes anserine tendinopathy, *MPQ* McGill pain questionnaire, *PI* patellar intumesce, *KOS-ADLS* Knee Outcome Survey-activities of Daily Living Scale, *ACL* anterior cruciate ligament, *LFS* Lysholm functional score, *MBS* Modified Blazina scale, *IKDC* International Knee Documentation Committee, *CPM* continuous passive motion, *LCSI* local corticosteroid injection, *HSS* Hospital for Special Surgery Knee score, *IPFP* infrapatellar fat pad, *ROM* range of motion

Among the 19 included RCTs, 6 used ESWT as monotherapy [16, 53, 80, 83, 88, 91], 1 used acupuncture therapy as adjunctive therapy [90], and 12 employed different types of cointerventions that included physiotherapy, acupuncture therapy, exercise training, manual therapy, and pharmacological medication [17, 48, 51, 52, 81, 82, 84–87, 89, 92]. Moreover, 9 RCTs reported an FU duration of 6 or 12 months [51, 52, 80, 82, 84–88], whereas the remaining 10 reported a short-term FU of ≤3 months [16, 17, 48, 53, 81, 83, 89–92].

Regarding the comparative alternatives administered to their control group, 9 RCTs used sham or no ESWT application [17, 48, 51, 81, 83, 84, 87, 89, 92], whereas 12 RCTs with a comparison control design used either noninvasive (conservative treatment [51, 52, 80, 81, 88–91] and specifically prescribed exercise training [51, 86]) or invasive (injection treatment [85] and acupuncture [53, 82]) treatment as ESWT alternatives.

The ESWT parameters and treatment protocols employed are summarized in Table 3. Of the eight RCTs that used FoSWT, five applied high-energy FoSWT [51, 84–87] and three applied low-energy FoSWT [16, 48, 83]. Of the 11 RCTs that used RaSWT, 5 employed high-energy RaSWT [52, 82, 88–90] and 6 employed low-energy RaSWT [17, 53, 80, 81, 91, 92]. Of all 19 RCTs, 18 applied an ESWT protocol comprising three to six treatment sessions over an intervention duration of 2–6 weeks [16, 17, 48, 52, 53, 80–85, 87–92], whereas one used a single ESWT session [86]. During ESWT sessions, local anesthesia was not administered at the treatment site in all included RCTs, except one, in which ESWT was applied immediately after surgery while patients were still under anesthesia [86].

**Table 3:** Type of wave characteristics, source of stimulation energy, and application parameters

Study author (year) [reference]	Energy generator	Source of energy	Device	Manufacturer	Shock wave treatment protocol							
					Application parameters (per session)					Total treatment sessions	Interval between sessions	Treatment duration (week)
					Rate (Hz)	EFD (mJ/mm^2^)	No. of impulses	TED^a^ (mJ/mm^2^)	Local anesthesia			
Chen (2014) [51]	Focused	Piezoelectric	Piezowave	Wolf, Germany	1–8	0.03–0.40	2000	60–800	Not used	6	1 week	6
Geng (2017) [90]	Radial	Pneumatic	LGT2500	Longest, China	NR	0.28	2000	560	Not used	4	1 week	4
Guan (2015) [80]	Radial	Pneumatic	DolorClast	EMS, Switzerland	5–7	0.05–0.075	2000–3000	100–225	Not used	3–5	1 week	3–5
Huang (2017) [88]	Radial	Pneumatic	NR	Xiangyu, China	12	0.18–0.31	2000	360–620	NR	5	1 week	5
Jiang (2016) [81]	Radial	NR	NR	NR	2–4	0.10–0.18	2500	100–625	Not used	6	2 days	2
Khosrawi (2017) [48]	Focused	NR	NR	NR	4	0.15	1500	225	Not used	3	1 week	3
Liu (2016) [82]	Radial	Pneumatic	DolorClast	EMS, Switzerland	10	0.21	2000	420	Not used	4	1 week	4
Taunton (2003) [83]	Focused	Electromagnetic	Sonocur	Siemens, USA	NR	0.17	2000	340	Not used	3–5	1 week	3–5
Thijs (2017) [84]	Focused	Piezoelectric	PiezoClast	EMS, Switzerland	1–2	0.20	1000	200	Not used	3	1 week	3
Vetrano (2013) [85]	Focused	Electromagnetic	Modulith SLK	Storz, Switzerland	NR	0.17–0.25	2400	408–600	Not used	3	2–3 days	2
Wang (2014) [86]	Focused	Electrohydraulic	OssaTron	HMT, Switzerland	NR	0.298	1500	447	Used	1		1
Weckström (2016) [52]	Radial	Pneumatic	Masterpuls MP 100	Storz, Switzerland	15	0.10–0.40	4600	460–1840	Not used	3	1 week	3
Wu (2009) [91]	Radial	Pneumatic	ESWO-AJ	EMS, Switzerland	1–15	0.10–0.12	2000	200–240	Not used	36	2 days	12
Wu (2016) [89]	Radial	Pneumatic	MP50	Storz, Switzerland	5–11	0.15–0.32	2000	300–640	Not used	4	5 days	3
Yang (2007) [16]	Focused	Electrohydraulic	HK.ESWO-AJ II	Wikkon, China	1	0.06–0.11	1000–2000	60–220	Not used	6	4 days	3–4
Zhang (2016) [92]	Radial	Pneumatic	MP100	Storz, Switzerland	10–15	0.08–0.15	2000–3000	300–640	Not used	18	2 days	6
Zhang (2017) [17]	Radial	Pneumatic	HK.ESWO-AJ II	Wikkon, China	8	0.11	1800–2000	60–220	Not used	8	3 days	4
Zhou (2015) [53]	Radial	Pneumatic	DolorClast	EMS, Switzerland	8–10	0.10–0.18	2000	200–360	Not used	5	4 days	3
Zwerver (2011) [87]	Focused	Piezoelectric	Piezowave	Wolf, Germany	4	0.10–0.58	2000	200–1160	Not used	3	1 week	3

^a^TED = EFD × number of shock wave impulses

*EMS* Electro Medical Systems, *HMT* High Medical Technology, *DMT* Dornier MedTech, *EFD* energy flux density, *TED* total energy dose (intensity × number of shock wave impulses), *NR* not reported

### Methodological quality of included RCTs

The methodological quality score of each RCT is listed in Tables 2 and 4. Regarding the cumulative PEDro score, interrater reliability was acceptable and the intraclass correlation coefficient was 0.98 (95% CI: 0.95–0.99, *P* < 0.001). The methodological quality of all the included RCTs was rated as high or medium, with a median (range) PEDro score of 6 (5–9).

**Table 4:** Summary of methodological quality based on the PEDro classification scale^c^

Study author (year) [reference]	Overall^a^	Eligibility criteria^b^	1	2	3	4	5	6	7	8	9	10
Chen (2014) [51]	7/10^d^		*X*	*X*	*X*				*X*	*X*	*X*	*X*
Geng (2017) [90]	6/10	*X*	*X*		*X*				*X*	*X*	*X*	*X*
Guan (2015) [80]	6/10	*X*	*X*		*X*				*X*	*X*	*X*	*X*
Huang (2017) [88]	6/10	*X*	*X*		*X*				*X*	*X*	*X*	*X*
Jiang (2016) [81]	6/10	*X*	*X*		*X*				*X*	*X*	*X*	*X*
Khosrawi (2017) [48]	8/10	*X*	*X*		*X*	*X*		*X*	*X*	*X*	*X*	*X*
Liu (2016) [82]	6/10	*X*	*X*		*X*				*X*	*X*	*X*	*X*
Taunton (2003) [83]	5/10^d^	*X*	*X*		*X*	*X*			*X*		*X*	
Thijs (2017) [84]	9/10	*X*	*X*	*X*	*X*	*X*		*X*	*X*	*X*	*X*	*X*
Vetrano (2013) [85]	7/10	*X*	*X*		*X*			*X*	*X*	*X*	*X*	*X*
Wang (2014) [86]	8/10	*X*	*X*	*X*	*X*			*X*	*X*	*X*	*X*	*X*
Weckström (2016) [52]	6/10	*X*	*X*	*X*	*X*				*X*		*X*	*X*
Wu (2009) [91]	6/10	*X*	*X*		*X*				*X*	*X*	*X*	*X*
Wu (2016) [89]	6/10	*X*	*X*		*X*				*X*	*X*	*X*	*X*
Yang (2007) [16]	5/10	*X*	*X*		*X*				*X*		*X*	*X*
Zhang (2016) [92]	6/10	*X*	*X*		*X*				*X*	*X*	*X*	*X*
Zhang (2017) [17]	6/10	*X*	*X*		*X*				*X*	*X*	*X*	*X*
Zhou (2015) [53]	6/10	*X*	*X*		*X*				*X*	*X*	*X*	*X*
Zwerver (2011) [87]	9/10	*X*	*X*	*X*	*X*	*X*		*X*	*X*	*X*	*X*	*X*

*PEDro* Physiotherapy Evidence Database

^a^Points of methodological quality are denoted as “*X*” for fulfilled criteria

^b^Not used to calculate the total score

^c^PEDro classification scale: 1 = random allocation, 2 = concealed allocation, 3 = similarity at the baseline, 4 = subject blinding, 5 = therapist blinding, 6 = assessor blinding, 7 = more than 85% follow-up for at least one key outcome, 8 = intention-to-treat analysis, 9 = between-group statistical comparison for at least one key outcome, 10 = point and variability measures for at least one key outcome. Methodological quality: high, ≥7 points; medium, 4–6 points; low, ≤3 points

^d^Score was determined by a third assessor

### Risk of bias of included RCTs

**Fig. 2:**
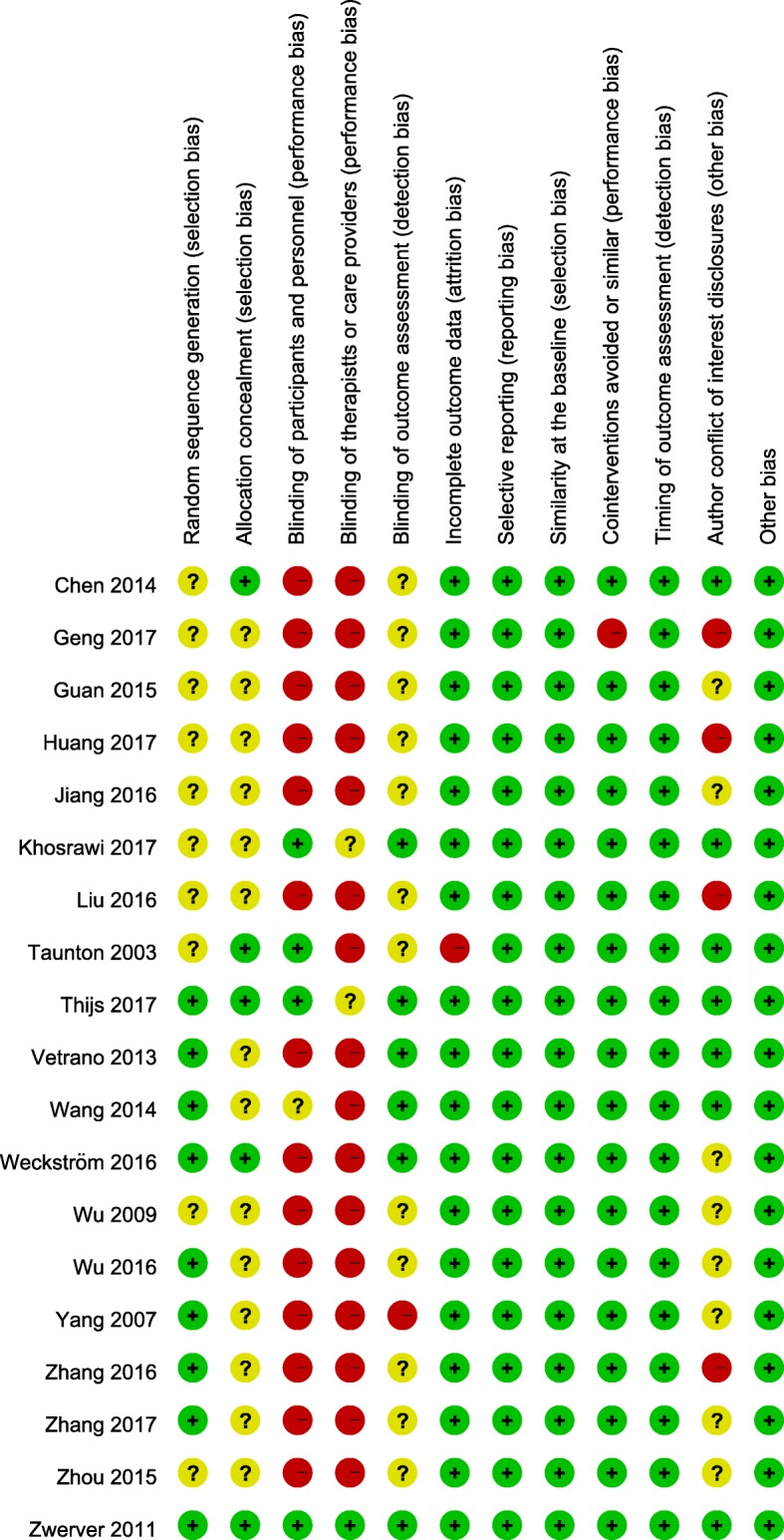
Risk of bias summary: review authors’ judgements about each risk of bias item for each included study

**Fig. 3:**
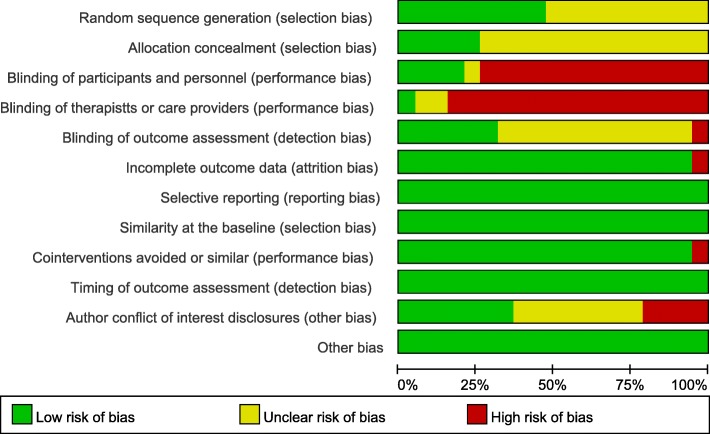
Risk of bias graph: review authors’ judgements about each risk of bias item presented as percentages across all included studies

Figure 2 shows details on each risk of bias item in each included RCT, as judged by the reviewing authors, and Fig. 3 provides an overall summary across the included RCTs. Selection, blinding, and attrition biases were considered to have caused the greatest risks of bias in the included RCTs.

#### Selection bias

Insufficient information on random sequence generation and allocation concealment led to selection bias in the included RCTs. Less than half of the included RCTs reported the randomization procedure [16, 17, 84–87, 89, 92] and concealed allocation [51, 52, 83, 84, 87] employed.

#### Performance bias

Difficulty in blinding participants and therapists (or care providers) when administering ESWT interventions with nonplacebo controls were deemed the major sources of performance bias in the included RCTs. The risk of performance bias was considered high in 14 [16, 17, 51–53, 80–82, 85, 88–92] and 16 [16, 17, 51–53, 80–83, 85, 86, 88–92] RCTs because participants and therapists were not blinded, respectively. One RCT applied ESWT immediately after ACL reconstruction surgery under the same anesthesia [86], which enabled masking of the group allocation to the patients while standard postoperative rehabilitation was performed [93]; however, because of the lack of information about whether the patients were blinded for group allocation in this RCT, its risk of bias was considered unclear.

#### Attrition bias

The assessor was blinded in six RCTs [48, 52, 84–87], and one RCT clearly declared that the assessors were not blinded [16]. However, the remaining 12 RCTs [17, 51, 53, 80–83, 88–92] did not mention blinding of the assessors.

#### Outcome reporting bias

All RCTs completely reported the results of all outcome measures described in the Methods section, including the pain score, patient-reported functional recovery, and performance-based measured outcomes (Table 2).

#### Agenda bias

Information on funding sources and authors’ conflict of interest disclosures is summarized in Table 2 and Additional file 2. Of the 19 included RCTs, nine were funded by one or more funding sources [48, 51, 82, 83, 86–88, 90, 92], whereas the remaining 10 did not report their funding source [16, 17, 52, 53, 80, 81, 84, 85, 89, 91]. Eight RCTs provided conflict of interest disclosures, of which two declared conflicts [83, 87] and the remaining five declared absence of conflicts [48, 51, 84–86] (Additional file 2: Table S2).

#### Publication bias

**Fig. 4:**
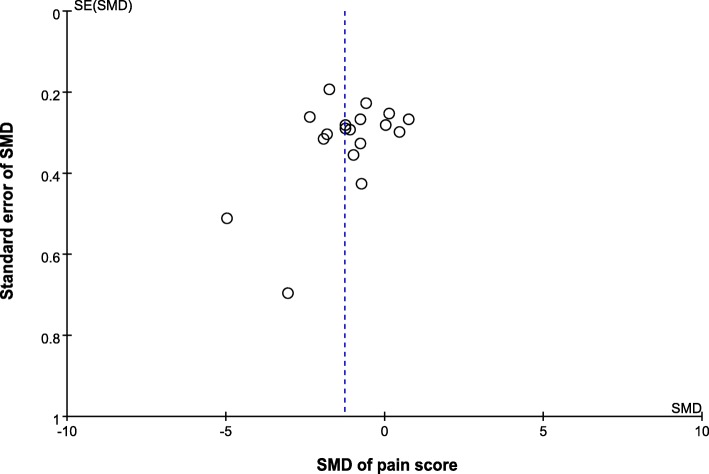
Funnel plot of standard mean difference (SMD) versus standard error (SE). The SMDs of the pain score are plotted on the *x*-axis, and the standard error of the SMD is plotted on the *y*-axis. The vertical dotted line indicates the mean value of the SMDs. Visual inspection of the funnel plot of the SMDs of the pain score did not reveal substantial asymmetry. Egger’s linear regression test indicated no evidence of reporting bias among the studies (*t* = − 2.03; *P* = 0.06)

Visual inspection of the funnel plots of pain reduction did not reveal substantial asymmetry (Fig. 4). Egger’s linear regression test also indicated no evidence of reporting bias among the trials (*t* = − 2.03; *P* = 0.06).

### Success or improvement rate

In total, 16 RCTs reported categorical data for pain and general outcomes (Table 2) [16, 17, 48, 52, 53, 82–92]. The treatment success rates (TSRs) for pain severity and global outcomes were mostly assessed using a Likert scale [57, 58] and were reported by nine RCTs [16, 17, 53, 82, 84, 89–92]. In addition, seven RCTs reported the proportions of patients who experienced pain relief and self-reported improved symptoms after ESWT [48, 52, 83, 85–88].

**Fig. 5:**
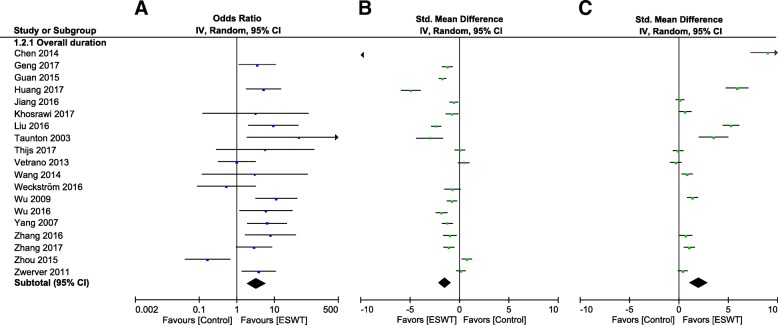
Forest plot of clinical efficacy of extracorporeal shock wave therapy (ESWT). Effect of ESWT on **a** treatment success rate, **b** pain reduction, and **c** functional outcome over overall follow-up duration. The horizontal line links the lower and upper limits of the 95% CI of this effect. The combined effects are plotted using black diamonds. 95% CI = 95% confidence interval; Random = random-effects model; Std. = standard. Details of each comparison are presented in Additional file 3: Figure S1, Additional file 5: Figure S3, and Additional file 7: Figure S5

There was moderate evidence from 16 RCTs [16, 17, 48, 52, 53, 82–92] (842 patients) that general ESWT yielded higher TSRs than did the placebo or active control (OR: 3.36, 95% CI: 1.84–6.12, *P* < 0.0001, *I*^2^ = 60%), regardless of the FU duration, shock-wave type, or application level (Fig. 5a and Additional file 3).

**Fig. 6:**
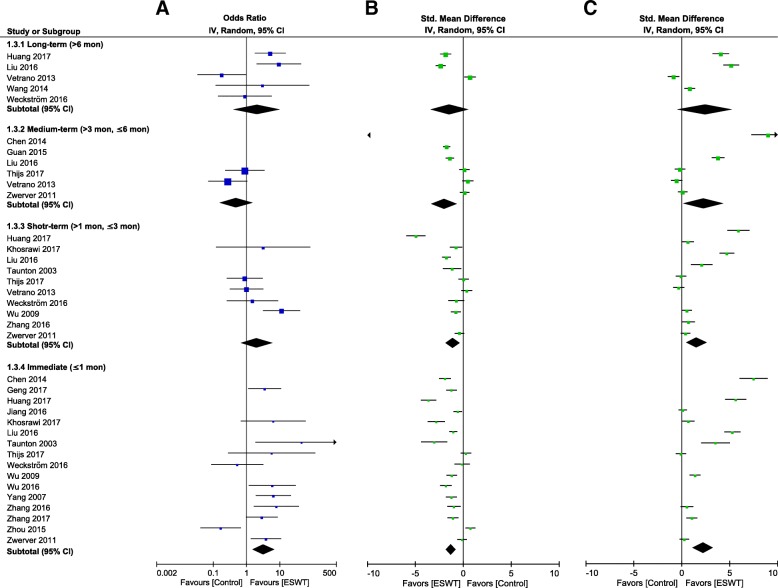
Forest plot of clinical efficacy of extracorporeal shock wave therapy (ESWT). Effect of ESWT on a treatment success rate, **b** pain reduction, and **c** functional outcome at each follow-up time point. The horizontal line links the lower and upper limits of the 95% CI of this effect. The combined effects are plotted using black diamonds. 95% CI = 95% confidence interval; Random = random-effects model; Std. = standard. Details of each comparison are presented in Additional file 4: Figure S2, Additional file 6: Figure S4, and Additional file 8: Figure S6

Subgroup analysis according to FU duration (Fig. 6a and Additional file 4) revealed moderate evidence from 11 RCTs [16, 17, 48, 52, 53, 83, 84, 87, 89, 90, 92] (518 patients) that at the immediate FU, general ESWT had a higher pooled OR for the TSR than the comparison control (OR: 3.09, 95% CI: 1.43–6.69, *P* = 0.004, *I*^2^ = 63%). General ESWT had no significant effect on the TSR at short-, medium-, and long-term FU assessments. Another subgroup analysis according to shock-wave type (Table 5) showed moderate evidence from 9 RCTs [17, 52, 53, 82, 88–92] (518 patients) that RaSWT had significant effects on the TSR at short-term, medium-term, and long-term FU assessments, with an overall pooled OR of 3.11 (*P* = 0.01, *I*^2^ = 73%), whereas FoSWT had significant effects only at the immediate FU, with an overall pooled OR of 3.28 (*P* = 0.001, *I*^2^ = 24%; LoE, strong; 7 RCTs [16, 48, 83–87], 324 patients).

**Table 5:** Summary of subgroup analysis results^a^

Subgroups	Treatment success rate					Pain score reduction					Patient-reported functional improvement				
	Trials (patient), *n*	OR	(95% CI)	*P* value	*I*^2^ (%), LoE^d^	Trials (patient), *n*	SMD	(95% CI)	*P* value	*I*^2^ (%), LoE^d^	Trials (patient), *n*	SMD	(95% CI)	*P* value	*I*^2^ (%), LoE^d^
Follow-up duration															
Focused ESWT															
Overall	7 (324)	3.28	(1.79, 6.02)^b^	0.001	25, S	7 (337)	−2.01	(−3.31, −0.71)^c^	0.002	96, M	7 (333)	1.08	(0.19, 1.97)^c^	0.02	92, M
> 6 months	2 (99)	0.33	(0.07, 1.45)^b^	0.14	56, C	1 (46)	0.71	(0.11, 1.31)	0.02	NA, M	2 (99)	−0.01	(−1.66, 1.65)^c^	1.00	94, C
> 3 months, ≤6 months	2 (87)	0.49	(0.19, 1.29)^b^	0.15	31, C	4 (220)	−2.74	(−4.85, −0.62)^c^	0.01	97, M	4 (220)	1.81	(−0.14, 3.77)^c^	0.07	97, C
> 1 month, ≤3 months	3 (129)	1.02	(0.45, 2.32)^b^	0.96	0, C	5 (220)	−0.31	(−0.78, 0.16)^c^	0.19	65, C	5 (220)	0.42	(−0.17, 1.00)^c^	0.16	76, C
≤ 1 month	5 (225)	5.53	(2.71, 11.25)^b^	< 0.00001	0, S	6 (291)	− 1.39	(−2.37, −0.41)^c^	0.005	92, M	5 (234)	2.22	(0.51, 3.92)^c^	0.01	96, M
Radial ESWT															
Overall	9 (518)	3.11	(1.31, 7.38)^c^	0.01	73, M	11 (747)	−1.36	(−2.02, −0.71)^c^	< 0.0001	93, L	6 (395)	2.56	(0.92, 4.19)^c^	0.002	97, L
> 6 months	3 (185)	4.35	(1.96, 9.63)^b^	0.0003	49, M	2 (165)	−2.13	(−2.52, −1.74)^b^	< 0.00001	42, M	2 (165)	4.64	(4.04, 5.25)^b^	< 0.00001	68, M
> 3 months, ≤6 months	0	NA		NA	NA	2 (246)	−1.59	(− 1.88, − 1.30)^b^	< 0.00001	20, M	1 (100)	3.79	(3.12, 4.45)	< 0.00001	NA, L
> 1 month, ≤3 months	2 (80)	5.60	(1.97, 15.86)^b^	0.001	69, M	4 (249)	−2.00	(−3.41, −0.58)^c^	0.006	95, L	4 (261)	2.93	(0.48, 5.37)^c^	0.02	98, L
≤ 1 month	6 (293)	1.88	(0.59, 5.98)^c^	0.28	75, C	10 (601)	−1.07	(−1.67, −0.47)^c^	0.0005	91, L	6 (395)	2.29	(0.73, 3.84)^c^	0.004	97, L
Energy level (EFD)															
Focused ESWT															
≥ 0.2 mJ/mm^2^	4 (211)	2.25	(1.08, 4.49)^b^	0.03	8, S	4 (220)	−2.94	(−5.05, −0.82)^c^	0.006	97, M	5 (273)	1.65	(0.21, 3.10)^c^	0.02	96, M
< 0.2 mJ/mm^2^	3 (113)	7.39	(2.52, 21.67)^b^	0.0003	0, M	3 (117)	−1.47	(−2.42, −0.53)^c^	0.002	77, M	2 (60)	1.99	(−0.82, 4.81)^c^	0.17	92, C
Radial ESWT															
≥ 0.2 mJ/mm^2^	5 (308)	3.98	(2.18, 7.29)^b^	< 0.00001	40, M	5 (311)	−2.17	(−3.23, −1.11)^c^	< 0.0001	92, L	2 (165)	5.49	(4.81, 6.17)^b^	< 0.00001	0, M
< 0.2 mJ/mm^2^	4 (210)	2.55	(0.43, 15.17)^c^	0.30	86, C	6 (436)	−0.73	(−1.44, −0.02)^c^	0.04	91, L	4 (230)	0.80	(0.19, 1.40)^c^	0.01	79, L
Intervention duration															
Focused ESWT															
≥ 1 month	2 (73)	7.89	(2.61, 23.88)^b^	0.0003	0, M	3 (137)	−3.13	(−5.70, −0.56)^c^	0.02	95, M	2 (80)	6.24	(0.86, 11.62)^c^	0.02	95, M
< 1 month	5 (251)	1.52	(0.80, 2.87)^b^	0.20	38, C	4 (200)	−0.17	(−0.45, 0.11)^b^	0.23	61, C	5 (253)	0.51	(−0.17, 1.19)^c^	0.14	88, C
Radial ESWT															
≥ 1 month	6 (375)	5.32	(3.20, 8.83)^b^	< 0.00001	0, M	7 (521)	− 1.80	(− 2.52, −1.08)^c^	< 0.00001	91, L	5 (315)	1.99	(1.68, 2.30)^c^	< 0.00001	97, L
< 1 month	3 (143)	0.78	(0.09, 6.69)^c^	0.82	81, C	4 (226)	−0.58	(−1.64, 0.48)^c^	0.29	93, C	1 (80)	0.11	(−0.33, 0.54)	0.64	NA, C
Control group type															
Focused ESWT															
Placebo	5 (221)	4.61	(1.92, 11.08)^b^	0.0006	0, S	5 (234)	−3.22	(−5.14, −1.31)^c^	0.001	97, M	6 (287)	2.03	(0.70, 3.36)^c^	0.003	95, M
Noninvasive comparison control	1 (57)	6.40	(1.89, 21.68)	0.003	NA, L	2 (117)	−2.07	(−3.73, −0.41)^c^	0.01	92, M	1 (60)	6.98	(5.59, 8.36)	< 0.00001	NA, M
Invasive comparison control	1 (46)	1.00	(0.31, 3.18)	1.0	NA, C	1 (46)	0.36	(−0.22, 0.95)	0.22	NA, C	1 (46)	−0.32	(−0.90, 0.26)	0.28	NA, C
Radial ESWT															
Placebo	3 (152)	4.41	(2.00, 9.71)^b^	0.0002	0, M	4 (232)	−1.14	(−1.42, −0.86)^b^	< 0.00001	56, M	3 (170)	0.70	(0.02, 1.39)^c^	0.04	77, L
Noninvasive comparison control	4 (206)	4.17	(2.23, 7.81)^b^	< 0.00001	61, M	5 (355)	−1.81	(−2.83, −0.80)^c^	0.0005	93, L	2 (125)	3.62	(−0.81, 8.04)^c^	0.11	98, C
Invasive comparison control	2 (160)	1.23	(0.02, 63.55)^c^	0.92	93, C	2 (160)	−0.80	(−3.85, 2.24)^c^	0.61	99, C	1 (100)	5.27	(4.43, 6.12)	< 0.00001	NA, L
Treated populations															
Focused ESWT															
Athlete	3 (124)	2.47	(1.16, 5.27)^b^	0.02	68, M	3 (128)	−0.84	(−2.16, 0.47)^c^	0.21	90, C	3 (128)	0.97	(−0.42, 2.37)^c^	0.17	91, C
Nonathlete	4 (200)	5.47	(1.98, 15.11)^b^	0.001	0, S	4 (209)	−3.61	(−5.86, −1.35)^c^	0.002	97, M	4 (205)	2.35	(0.38, 4.31)^c^	0.02	97, M
Radial ESWT															
Athlete	4 (285)	3.10	(0.50, 19.30)^c^	0.23	87, C	4 (285)	−1.79	(−3.73, 0.15)^c^	0.07	98, C	3 (225)	4.16	(1.09, 7.24)^c^	0.008	98, L
Nonathlete	5 (233)	3.22	(1.75, 5.94)^b^	0.0002	32, M	7 (462)	−1.24	(−1.57, −0.91)^c^	< 0.00001	60, L	3 (170)	0.59	(0.01, 1.18)^c^	0.05	72, L
Treated disease															
Focused ESWT															
Tendinopathy	5 (214)	3.62	(1.28, 5.36)^b^	0.008	38, S	6 (280)	−2.29	(−3.84, −0.75)^c^	0.004	96, M	6 (280)	1.14	(0.06, 2.21)^c^	0.04	94, M
Other KSTDs	2 (110)	5.83	(1.86, 18.26)^b^	0.002	0, M	1 (57)	−1.24	(−1.81, −0.67)	< 0.0001	NA, L	1 (53)	0.84	(0.27, 1.40)	0.004	NA, M
Radial ESWT															
Tendinopathy	5 (306)	4.67	(2.61, 8.36)^b^	< 0.00001	54, M	7 (535)	−1.70	(−2.48, − 0.92)^c^	< 0.0001	93, L	4 (305)	3.47	(0.78, 6.16)^c^	0.01	98, L
Other KSTDs	4 (212)	2.13	(0.40, 11.43)^c^	0.38	83, C	4 (212)	−0.77	(−1.92, 0.38)^c^	0.19	93, C	2 (90)	0.91	(0.47, 1.34)^b^	< 0.0001	0, M
Cointervention design															
Focused ESWT															
Monotherapy	1 (16)	45.00	(1.83, 1104.64)	0.0002	NA, L	2 (80)	−5.17	(−6.43, −3.91)^c^	< 0.0001	98, M	2 (80)	6.24	(0.86, 11.62)^c^	0.02	95, M
Cointervention	6 (308)	2.98	(1.61, 5.52)^b^	0.0005	6, S	5 (257)	−0.26	(−0.87, 0.35)^c^	0.41	83, C	5 (253)	0.29	(−0.12, 0.71)^c^	0.17	63, C
Radial ESWT															
Monotherapy	3 (185)	2.18	(0.21, 22.89)^c^	0.52	91, C	4 (331)	−1.62	(−3.32, 0.08)^c^	0.06	97, C	2 (125)	3.99	(0.31, 7.68)^c^	0.03	97, L
Cointervention	6 (333)	3.72	(2.11, 6.57)^b^	< 0.00001	33, M	7 (416)	−1.26	(−1.78, −0.75)^c^	< 0.00001	82, L	4 (270)	2.11	(0.02, 4.21)^c^	0.05	98, L

^a^*OR* odds ratio, *I*^2^ heterogeneity, *LoE* level of evidence, *SMD* standard mean difference, *NA* not applicable, *EFD* energy flux density, *ESWT* extracorporeal shock wave therapy, *KSTDs* knee soft tissue disorders

^b^Fixed-effects model

^c^Random-effects model

^d^Level of evidence: Strong (S), Moderate (M), Limited (L), Very limited (V), Conflicting (C)

Subgroup analysis according to shock-wave type, dosage level, and intervention duration (Table 5) revealed moderate evidence that RaSWT administered at high energy (5 RCTs [52, 82, 88–90], 308 patients; OR: 3.98, *P* < 0.00001, *I*^2^ = 40%) and over a long intervention period (6 RCTs [17, 82, 88, 90–92], 375 patients; OR: 5.32, *P* < 0.00001, *I*^2^ = 0%) resulted in a significantly higher TSR than the corresponding control, as indicated by the higher pooled ORs; similar results were noted for FoSWT. Furthermore, low-energy FoSWT also had a higher pooled OR for TSR than its control (3 RCTs [16, 48, 83], 113 patients; OR: 5.32, *P* < 0.00001, *I*^2^ = 0%; LoE, moderate).

Another subgroup analysis according to control intervention showed that FoSWT as well as RaSWT resulted in a higher TSR than did the placebo control (5 RCTs [48, 83, 84, 86, 87], 221 patients; ORs 4.61, *P* = 0.0006, *I*^2^ = 0%; LoE, strong) and noninvasive comparisons (1 RCT [16], 57 patients; ORs 6.40, *P* = 0.003; LoE, limited; Table 5). No difference was noted in the TSR for pain relief between FoSWT and the invasive comparison control; similar results were obtained for RaSWT.

FoSWT resulted in significantly higher TSRs in both athletes (3 RCTs [83, 85, 87], 124 patients; OR: 2.47, *P* = 0.02, *I*^2^ = 68%; LoE, moderate) and nonathletes (4 RCTs [16, 48, 84, 86], 200 patients; OR: 5.47, *P* = 0.001, *I*^2^ = 0%; LoE, strong) than in their control peers (Table 5). However, RaSWT exhibited a significant effect on TSRs in nonathletes alone (5 RCTs [17, 52, 89, 90, 92], 233 patients; OR: 3.22, *P* = 0.0002, *I*^2^ = 32%; LoE, moderate).

In patients with tendinopathies, both FoSWT and RaSWT exerted significant effects on TSRs, with pooled ORs of 3.62 (*P* = 0.008, *I*^2^ = 38%; 5 RCTs [48, 83–85, 87], 214 patients; LoE, strong) and 4.67 (*P* < 0.00001, *I*^2^ = 54%; 5 RCTs [52, 82, 88, 90, 91], 306 patients; LoE, moderate), respectively (Table 5). In patients with other KSTDs, FoSWT employed to treat ACL injury [86] and posttraumatic knee stiffness [16] had a significant effect on the TSRs, with a pooled OR of 5.83 (*P* = 0.002, *I*^2^ = 0%; LoE, moderate). However, in four RCTs, using RaSWT to treat ACL injury [89], traumatic knee synovitis [92], posttraumatic knee stiffness [17], and infrapatellar fat pad injury [53] did not result in significantly high TSRs (Table 5). Nevertheless, after excluding the RCT with an invasive comparison control [53], RaSWT had a significant effect on TSR among patients with other KSTDs (OR: 4.41, 95% CI: 2.00–9.71, *P* = 0.0002, *I*^2^ = 0%; LoE, moderate).

When applied with a monotherapy [83] and cointervention [16, 48, 84–87] design, FoSWT exerted a significant effect on TSRs (185 patients, OR: 11.73, *P* = 0.0002, *I*^2^ = 0%, LoE, limited and 308 patients, OR: 2.98, *P* = 0.0005, *I*^2^ = 6%, LoE, strong, respectively; Table 5). However, in the subgroup of RaSWT, only the six RCTs [17, 48, 52, 82, 91, 92] (333 patients) with a cointervention design showed significant effects on TSRs (OR: 4.53, *P* < 0.00001, *I*^2^ = 48%; LoE, moderate).

### Effect on pain reduction

Eighteen RCTs assessed pain severity using the VAS [16, 17, 48, 51–53, 80–85, 87–92]. All pain severity data were transformed into 0–100-mm continuous data. Analysis of transformed pain scores revealed moderate evidence with large effect from 18 RCTs [16, 17, 48, 51–53, 80–85, 87–92] (1084 patients) that pain was significantly ameliorated after ESWT, with an overall pooled SMD of − 1.49 (95% CI: − 2.11 to − 0.87, *P* < 0.00001, *I*^2^ = 95%) compared with the control group, regardless of the FU duration, shock-wave type, application level, or control intervention type (Fig. 5b and Additional file 5).

Subgroup analysis according to FU duration (Fig. 6b and Additional file 6) indicated moderate evidence with medium effect from 16 RCTs [16, 17, 48, 51–53, 81–84, 87–92] (892 patients) that general ESWT resulted in immediate pain relief, with an SMD of − 1.18 (95% CI: − 1.67 to − 0.68, *P* < 0.00001, *I*^2^ = 91%), regardless of the shock-wave type, dosage level, or control intervention type. Similar results were obtained for short-term (10 RCTs [48, 52, 82–85, 87, 88, 91, 92], 469 patients; SMD: − 1.07, 95% CI: − 1.84 to − 0.31, *P* = 0.006, *I*^2^ = 93%; LoE, moderate) and medium-term (6 RCTs [51, 80, 82, 84, 85, 87], 466 patients; SMD: − 1.98, 95% CI: − 3.32 to − 0.64, *P* = 0.004, *I*^2^ = 97%; LoE, moderate) FUs. Another subgroup analysis according to shock-wave type (Table 5) revealed that RaSWT had significant effects on pain reduction at each FU, with an overall pooled SMD of − 1.36 (*P* < 0.0001, *I*^2^ = 93%; 11 RCTs [17, 52, 53, 80–82, 88–92], 747 patients; LoE, limited). FoSWT also had significant effects on pain reduction at all FU durations except the short-term FU, with an overall pooled SMD of − 2.01 (*P* = 0.002, *I*^2^ = 96%; 7 RCTs [16, 48, 51, 83–85, 87], 337 patients; LoE, moderate).

Subgroup analysis according to shock-wave type and application level revealed modrate evidence with large effects that high-energy (4 RCTs [51, 84, 85, 87], 220 patients; SMD: − 2.94, 95% CI: − 5.05 to − 0.82, *P* = 0.006, *I*^2^ = 97%) and low-energy (3 RCTs [16, 48, 83], 117 patients; SMD: − 1.47, 95% CI: − 2.42 to − 0.53, *P* = 0.002, *I*^2^ = 77%) FoSWT as well as long intervention duration (3 RCTs [16, 51, 83], 137 patients; SMD: − 3.13, 95% CI: − 5.70 to − 0.56, *P* = 0.02, *I*^2^ = 95%) exerted significant effects on pain reduction (Table 5). Similar results were obtained for RaSWT. Neither FoSWT nor RaSWT with an intervention duration of < 1 month exerted a significant effect on pain reduction.

Compared with the placebo control, there were moderate evidences that FoSWT and RaSWT had a significant effect on pain reduction (5 RCTs [48, 51, 83, 84, 87], 234 patients, SMD: − 3.22, *P* = 0.001, *I*^2^ = 97% and 4 RCTs [17, 81, 89, 92], 232 patients, SMD: − 1.14, *P* < 0.00001, *I*^2^ = 56%, respectively); similar results were noted in the comparison with the noninvasive controls (Table 5). Compared with the invasive comparison controls, FoSWT and RaSWT did not have a significant effect on pain reduction.

There was moderate evidence with large effect from 4 RCTs [16, 48, 51, 84] (209 patients) that nonathletes experienced significant pain reduction after FoSWT (SMD: − 3.61, *P* = 0.002, *I*^2^ = 97%) but athletes did not (Table 5). However, after excluding RCTs with a short intervention period [85, 87], we observed a significant effect in athletes (SMD: − 3.03, *P* < 0.0001); similar results were noted for RaSWT.

In patients with tendinopathies, both FoSWT (6 RCTs [48, 51, 83–85, 87], 280 patients) and RaSWT (7 RCTs [52, 80–82, 88, 90, 91], 535 patients) had a significant effect on pain reduction, with pooled SMDs of − 2.29 (*P* = 0.004, *I*^2^ = 96%; LoE, moderate) and − 1.70 (*P* < 0.0001, *I*^2^ = 93%; LoE, limited), respectively (Table 5). In patients with other KSTDs, FoSWT—employed by only one RCT [16] (57 patients) to treat posttraumatic knee stiffness—exerted a significant effect on pain reduction (SMD: − 1.24, *P* < 0.0001; LoE, limited); by contrast, RaSWT—employed by four RCTs to treat ACL injury [89], traumatic knee synovitis [92], posttraumatic knee stiffness [17], and infrapatellar fat pad injury [53] in these patients—did not exert a significant effect (Table 5). Moreover, after excluding an RCT that administered an invasive comparison control [53], we observed that RaSWT had a significant effect on pain reduction in these patients (SMD: − 1.31, 95% CI: − 1.67 to − 0.96, *P* < 0.0001, *I*^2^ = 53%).

There was moderate evidence with large effect from two RCTs [51, 83] (80 patients) that FoSWT employed as monotherapy had a significant effect on pain reduction, with a pooled SMD of − 5.17 (*P* < 0.0001, *I*^2^ = 98%), whereas FoSWT administered with a cointervention, as occurred in five other RCTs [16, 48, 84, 85, 87], did not (Table 5). In contrast to the results for FoSWT, RaSWT employed as monotherapy had no significant effect on pain reduction; however, that with a cointervention, as occurred in seven RCTs [17, 52, 81, 82, 89, 90, 92] (416 patients), did (SMD: − 1.26, *P* < 0.00001, *I*^2^ = 82%; LoE, limited).

### Effect on patient-reported functional outcomes

Thirteen RCTs used patient-report questionnaires to evaluate disability, functional mobility, and general outcomes (Table 2) [17, 48, 51, 81–88, 91, 92]. In particular, six RCTs [82–85, 87, 88] used the Victorian Institute of Sport Assessment-Patella questionnaire [59], one [86] used the International Knee Documentation Committee subjective score [94], one [51] used Lequesne’s index [95], two [86, 92] used the Lysholm functional score [94], two [48, 91] used the McGill pain questionnaire [96], one [17] used the Hospital for Special Surgery Knee score [97], and one [81] used the Knee Outcome Survey-Activities of Daily Living Scale [98]. Combined analysis revealed moderate evidence with large effect (13 RCTs [17, 48, 51, 81–88, 91, 92], 728 patients; SMD of 2.03 (95% CI: 1.09–2.96, *P* < 0.0001, *I*^2^ = 96%), favoring general ESWT regardless of the FU duration, shock-wave type, application level, control intervention type, or treated population (Fig. 5c and Additional file 7).

Subgroup analysis according to the FU duration (Fig. 6c and Additional file 8: Figure S6) revealed that general ESWT had an immediate effect on functional outcomes, with an SMD of 2.24 (95% CI: 1.16–3.33, *P* < 0.0001, *I*^2^ = 97%; 11 RCTs [17, 48, 51, 81–84, 87, 88, 91, 92], 629 patients; LoE, moderate), regardless of the shock-wave type, dosage level, or control intervention type. Similar results were observed at short-term (9 RCTs [48, 82–85, 87, 88, 91, 92], 481 patients; SMD: 1.56, 95% CI: 0.46–2.67, *P* = 0.006, *I*^2^ = 96%; LoE, moderate) and medium-term (5 RCTs [51, 82, 84, 85, 87], 320 patients; SMD: 2.28, 95% CI: 0.20–4.35, *P* = 0.03, *I*^2^ = 98%; LoE, moderate) FUs. Another subgroup analysis according to shock-wave type (Table 5) showed limited evidence with large effect from 6 RCTs [17, 81, 82, 88, 91, 92] (395 patients) that RaSWT exerted significant effects on functional recovery at each FU, with an overall pooled SMD of 2.56 (*P* = 0.002, *I*^2^ = 97%). However, FoSWT exerted significant effects only for immediate FUs, with an overall pooled SMD of 1.08 (*P* = 0.02, *I*^2^ = 92%; 7 RCTs [48, 51, 83–87], 333 patients; LoE, moderate).

Subgroup analysis according to shock-wave type and energy level (Table 5) showed moderate evidences with large effects that FoSWT applications with high energy (5 RCTs [51, 84–87], 273 patients; SMD: 1.65, 95% CI: 0.21–3.10, *P* = 0.02, *I*^2^ = 96%) and long intervention duration (2 RCTs [51, 83], 80 patients; SMD: 6.24, 95% CI: 0.86–11.62, *P* = 0.02, *I*^2^ = 95%) had significant effects on pain relief; similar results were noted for RaSWT. Both FoSWT and RaSWT—used by five RCTs [48, 84–87] and one RCT [81], respectively—with a short intervention duration exerted nonsignificant pooled effects on pain reduction.

Compared with the placebo control (six RCTs [48, 51, 83, 84, 86, 87], 287 patients) and noninvasive (one RCT [51], 60 patients) comparisons, moderate evidences with large effects favoring FoSWT (SMD 2.03, *P* = 0.003, *I*^2^ = 95% and SMD 6.98, *P* < 0.00001, respectively) were observed; no difference was observed between FoSWT and the invasive comparison control, which is in contrast to the results for RaSWT (one RCT [82], 100 patients; SMD 5.27, *P* < 0.00001; LoE, limited; Table 5). In addition, with the placebo control, RaSWT exerted significant effects on function recovery (3 RCTs [17, 81, 92], 170 patients; SMD 0.70, *P* = 0.04; LoE, limited) but not with the noninvasive comparisons.

There was moderate efidence with large effect from four RCTs [48, 51, 84, 86] (205 patients) that FoSWT exerted significant effects on patient-reported functional outcomes in nonathletes (SMD: 2.35, *P* = 0.02, *I*^2^ = 97%) but not athletes (Table 5). However, after RCTs with short intervention duration [85, 87] were excluded, FoSWT exerted a significant effect in athletes (one RCT [83], 20 patients; SMD: 3.52; *P* < 0.00001; LoE, limited). Athletes (three RCTs [82, 88, 91], 225 patients; SMD: 4.16; *P* = 0.008, *I*^2^ = 98%; LoE, limited), as well as nonathletes (three RCTs [17, 81, 92], 170 patients; SMD: 0.59; *P* = 0.05, *I*^2^ = 72%; LoE, limited), showed significantly improved functional outcomes in response to RaSWT.

For patients with tendinopathies, both FoSWT (six RCTs [48, 51, 83–85, 87], 280 patients) and RaSWT (four RCTs [81, 82, 88, 91], 305 patients) had a significant effect on patient-reported functional outcomes, with pooled SMDs of 1.14 (*P* = 0.04, *I*^2^ = 94%; LoE, moderate) and 3.47 (*P* = 0.01, *I*^2^ = 98%; LoE, limited), respectively (Table 5). Similar results were obtained for patients with other KSTDs receiving FoSWT (one RCT [86], 53 patients; SMD: 0.84, *P* = 0.004; LoE, moderate) or RaSWT (two RCTs [17, 92], 90 patients; SMD: 0.91; *P* < 0.0001, *I*^2^ = 0%; LoE, moderate).

There was moderate evidence with large effect from two RCTs [51, 83] (80 patients) that FoSWT implemented as monotherapy exerted a significant effect on patient-reported functional recovery (SMD: 6.24; *P* = 0.02, *I*^2^ = 95%), whereas FoSWT administered with a cointervention, as occurred in five RCTs [48, 84–87], did not (Table 5). The effect on patient-reported functional recovery was similar in the RCTs that administered RaSWT as a monotherapy (two RCTs [88, 91], 125 patients; SMD: 3.99; *P* = 0.03, *I*^2^ = 97%; LoE, limited) and those with a cointervention design (four RCTs [17, 81, 82, 92], 270 patients; SMD: 2.11, *P* = 0.05, *I*^2^ = 98%; LoE, limited).

### Effect on performance-based functional outcomes

Only five RCTs used performance-based tests to evaluate functional recovery: the range of motion (ROM) measurement [16, 17, 51, 92] and the vertical jump test [83]. Four RCTs [16, 17, 51, 92] reported recovery in knee ROM and obtained moderate evidences, favoring FoSWT (two RCTs [16, 51], 117 patients) and RaSWT (two RCTs [17, 92], 90 patients), with consistent significant pooled SMDs of 2.61 (95% CI: 2.11–3.12, *P* < 0.00001, *I*^2^ = 0%) and 1.09 (95% CI: 0.64–1.53, *P* < 0.00001, *I*^2^ = 0%), respectively, regardless of the FU duration (Additional file 9). There was limited evidence with large effect from one RCT [83] (20 patients) that FoSW group exibited a significantly greater height in vertical jump test (SMD: 2.15; *P* = 0.0002, 95% CI: 1.00–3.30) compared with the placebo control group (Additional file 9).

### Side effects of ESWT

The adverse events and loss to FU in each included RCT are summarized in Additional file 10. In all included RCTs, no clinically relevant adverse events, side effects, or severe complications (e.g., hematomas, tendon rupture, and other abnormal musculoskeletal events) were reported after ESWT. Loss to FU in the FoSWT group occurred in four RCTs [51, 84, 85, 87], in which one to seven patients (3.2 to 31.8%) in the ESWT group withdrew from the study due to unknown reasons or reasons unrelated to the intervention. No patient was lost to FU in two FoSWT RCTs [16, 86], whereas the two other RCTs employing FoSWT [48, 83] did not provide information on adherence to shock-wave treatment. Compared with patients in the FoSWT group, no patient receiving RaSWT in 10 RCTs [17, 52, 53, 81, 82, 88–92] dropped out; however, one RCT using RaSWT [80] did not provide information on the number of patients lost to FU.

## Discussion

### Summarizing the evidence obtained in this meta-analysis

In this meta-analysis, we conducted a comprehensive search to select previous RCTs of the clinical efficacy of ESWT in patients with KSDTs. The results revealed significant moderate evidence of the safety and efficacy of general ESWT in increasing the TSR, reducing pain, enhancing patient-reported functional recovery, and improving performance-based functional outcomes in patients with KSTDs, regardless of the FU duration, shock-wave type, application level, control-intervention type, or treated population. Low-energy FoSWT may have higher efficacy in increasing the TSR and enhancing patient-reported functional outcomes than high-energy FoSWT. The reverse was the case for RaSWT. The intervention duration may have a higher influence on the efficacy of both RaSWT and FoSWT for KSTDs than the energy level.

### Superiority of different shock-wave types and application levels

The present study demonstrated the pooled effects of ESWT for KSTDs according to the shock-wave type, energy level in EFD, and intervention duration, in contrast to previous systemic reviews of the efficacy of ESWT for lower-extremity musculoskeletal disorders [36, 45–47, 99]. Previously, van der Worp et al. compared the effects of FoSWT and RaSWT on patellar tendinopathy, and the treatment protocol comprised a low energy level (0.12 mJ/mm^2^) and a short intervention period (3 weeks); they found no significant differences in the effects of FoSWT and RaSWT on the TSR and functional recovery at short-term (7 weeks) and medium-term (14 weeks) FU [100]. Król et al. revealed similar results for the effects of FoSWT and RaSWT on pain reduction at 3-, 6-, and 12-week FU time points in patients with elbow tendinopathy [101]. Compared with the aforementioned results, the present study that focused on KSTDs showed inconsistent results; that is, RaSWT exerted significant effects on the TSR and functional recovery at each FU time point, whereas FoSWT exerted significant effects only at immediate FU. Results of the present study may indicate that RaSWT is more likely to result in the highest treatment success or functional recovery than FoSWT. Nevertheless, this study also demonstrated that FoSWT and RaSWT with an application of a short intervention period had no difference in treatment efficacy. The discrepancy between the results of our meta-analysis and the findings of van der Worp [100] may be due to most RCTs included in our meta-analysis used a longer intervention period (> 3 weeks) and a higher EFD (> 0.12 mJ/mm^2^) than those used by van der Worp did. These differences in the intervention period and EFD further explain our findings regarding the difference in athletes’ responses to FoSWT and RaSWT. Athletes receiving FoSWT were mainly included from RCTs with short intervention periods, and those receiving RaSWT were mostly included from RCTs with long intervention periods. In addition, these differences also explain the difference in the effects of FoSWT on pain reduction and patient-reported functional recovery between RCTs with a monotherapy design and those with a cointervention design.

The influence of shock wave energy or dose on efficacy remains debatable. Previous studies have identified a dose-related effect on the treatment efficacy of ESWT. High-energy ESWT is recommended for treating calcified tendinitis [38, 102–104], whereas a low dose is more likely to result in the highest TSR and pain reduction for plantar fasciitis than medium or high doses [105, 106]. The inconsistency in the results of previous studies may arise from the inconsistent cutoff points set for low- and high-energy ESWT, which were set at 0.08 mJ/mm^2^ [102, 103, 105], 0.12 mJ/mm^2^ [38, 104, 106], and 0.33 mJ/mm^2^ [107] for low-energy ESWT and at 0.12 mJ/mm^2^ [38, 104], 0.28 mJ/mm^2^ [102, 103, 105, 106], and 0.78 mJ/mm^2^ [107] for high-energy ESWT, regardless of treated conditions. In the present study, we used an EFD value of 0.20 mJ/mm^2^ as the cutoff for low and high energy levels; the results reveal that compared with high-energy FoSWT, the low-energy FoSWT may exert greater effects on the TSR and functional recovery and may exert similar effects on pain reduction in patients with KSTDs. Contrary to the results of FoSWT, high-energy RaSWT showed significant efficacy for all outcomes, whereas low-energy RaSWT did not. Given that an EFD of < 0.2 mJ/mm^2^ has been identified as the optimal energy for FoSWT for tissue regeneration [30, 40, 108–110] and that low-energy RaSWT seems to have limited biological effects on human tendinopathy [33], results in this study may indicate the optimal use of low-energy FoSWT and high-energy RaSWT for enhancing clinical efficacy, particularly for the patients with KSTDs.

The other findings of this meta-analysis are as follows: (i) The intervention period may influence the efficacy of FoSWT or RaSWT. To date, few studies have analyzed various ESWT protocols based on the corresponding intervention periods. This meta-analysis demonstrated that an intervention period of ≥1 month exerted significantly effects on all outcomes favoring ESWT whereas a short intervention period (< 1 month) did not, regardless of ESWT type. This meta-analysis further identified no difference in the efficacy of FoSWT and RaSWT for KSTDs when both therapies were applied with a short intervention period in combination with either high or low EFD. The aforementioned results are supported by the results of previous studies, which showed that RaSWT with a short intervention period (< 1 month) exhibits efficacy similar to that of FoSWT with the same intervention period (< 1 month), regardless of the energy level [100, 101]. (ii) For treating KSTDs, both FoSWT and RaSWT had significant effects on the TSR versus their placebo control or noninvasive comparisons. Furthermore, FoSWT and RaSWT which are noninvasive therapies may be alternatives to such invasive interventions as local corticosteroid injection. However, in this meta-analysis, limited RCTs regarding the efficacy of ESWT versus invasive interventions were available. Thus, we could not obtain conclusive results in favor of ESWT over invasive interventions. Additional RCTs are required to determine the difference in efficacy between ESWT applications and invasive interventions. We further observed that the pooling RCTs with different type of controlled comparisons in the same subgroup may affect the efficacy of ESWT. For example, The subgroup including patients with other KSTDs than tendinopathy exhibited nonsignificant responses to RaSWT in terms of the TSR and pain reduction. The subgroup comprised patients from four RCTs of which only one conducted by Zhou et al. employed RaSWT versus an invasive intervention [53]. After the exclusion of Zhou’s study from meta-analyses, the results showed significant effects on the TSR and pain reduction favoring RaSWT, and heterogeneity was improved. (iii) Our meta-analysis indicated that low-energy FoSWT exerted higher effects on the TSR and patient-reported functional recovery than high-energy FoSWT, and the RaSWT showed an inverse case. The shock waves applied in FoSWT and RaSWT have different physical characteristics, and the original source of energy production differs between these therapies. The acoustic wave generated in FoSWT is transmitted into the deep tissue and centrally converges on the targeted tissue, whereas that generated in RaSWT radially penetrates the body [13, 34]. Based on the nature of energy transconduction, the two shock-wave types have different magnitudes of energy (i.e., EFD) at the same tissue depth; in addition, the FoSWT sequentially travels further and has a greater impact on deeper tissues, whereas the RaSWT has superficially maximal energy at its origin [11]. Therefore, it is reasonable that the energy level of RaSWT should be higher than that of FoSWT for producing the same pulse energy at deeper targeted tissues, which may explain the discrepancy in the efficacy of high-energy and low-energy FoSWT and RaSWT for KSTDs in this meta-analysis.

### Strengths and limitations

Compared with previous systemic reviews and meta-analyses of the efficacy of ESWT for knee orthopedic conditions [45–47], we included only RCTs to ensure level 1a evidence for therapy [111], and we included non-English trials [16, 17, 53, 80–82, 88–92, 112–115]. We also included RCTs involving soft tissue disorders other than patellar tendinopathy [16, 17, 48, 51–53, 80, 86, 89, 91, 92, 113]. Furthermore, we pooled comprehensive data to distinguish clinical efficacy levels at immediate, short-term, medium-term, and long-term FU, and we compared the clinical efficacy of different ESWT applications, namely different shock-wave types, application levels (i.e., energy in EFD and intervention duration), types of comparison controls, and treated populations (i.e., athletes and nonathletes). We performed comprehensive subgroup analyses to identify differences between different study designs (i.e., comparison types and patient types) and application levels (i.e., shock-wave type, energy level, and intervention duration).

Our meta-analysis has some limitations. First, not all types of KSTDs were included in this meta-analysis. Thus, the results may not be generalizable to other upper or lower limb conditions such as supraspinatus tendinopathy and Achilles’ tendinopathy. Second, although the data did not suggest substantial publication bias and suggested a significant effect size for pain reduction, favoring general ESWT, we observed heterogeneity across the included trials. The noted heterogeneity may be due to the varying designs and application protocols of the included RCTs. Third, other application parameters such as the rate of shocks (impulses per second, Hz), number of treatments, and interval between treatments, which may interfere with therapeutic response, were not considered in comparisons in this study [9, 42]. Fourth, most of the 20 RCTs included in this meta-analysis described patient-reported outcomes; only five RCTs reported performance-based functional outcomes including knee ROM [17, 51, 86, 92] and jump height in the vertical jump test [83]. In our meta-analysis, RCTs reporting other performance-based functional outcomes, such as muscle strength, balance, and mobility, were not available. Compared with patient-reported outcome measures, performance-based outcome measures can provide more objective information on physical function in patients with knee disorders [116–118]. Thus, more data on performance-based physical functional outcomes are required to differentiate the efficacy of various ESWT applications. Fifth, in this meta-analysis, high risks of selection, blinding, and performance biases were identified; other potential biases, including agenda bias and biases resulting from cointerventions and loss to FU, were also noted. Because nearly half of the included RCTs reported funding information, and had a cointervention design and because more drop-out events were reported for FoSWT than for RaSWT, the results of this meta-analysis should be interpreted with consideration of the aforementioned potential biases. Finally, other confounding factors, such as age, sex, participation in sports, physical activity level, work type, and rate of return to sports and work, which may have contributed to treatment efficacy, were not considered in the analysis of the TSR.

## Conclusions

### Findings

This study obtained moderate evidence that general ESWT significantly increases TSR, reduces pain, and improves functional recovery in patients with KSTDs, based on meta-analysis of RCTs with acceptable methodology quality (PEDro score ≥ 5/10) but high risks of potential selection, blinding, and performance biases. Additionally, this study provided limited to moderate evidence that both FoSWT and RaSWT with long intervention periods are superior to those with short intervention periods, regardless of the energy level. For long intervention periods, ESWTs can be ordered as follows in terms of their pooled effects on overall clinical outcomes for KSTDs: low-energy FoSWT, high-energy RaSWT, and high-energy FoSWT therapy. Furthermore, ESWT can be effectively performed with no severe adverse events other than a few minor side effects [101]. Both shock-wave therapies are worth considering in the treatment of soft tissue disorders, particularly KSTDs.

### Implications for clinical practice

Our findings can help clinicians in identifying alternatives to conventional management strategies of KSTDs for determining the optimal treatment strategy.

### Cautious application of ESWT for certain KSTDs

The generalizability of our findings is limited to the KSTDs reported in this meta-analysis. In addition, because our meta-analysis did not include sufficient RCTs involving KSTDs such as infrapatellar fat pad injury, traumatic knee synovitis, iliotibial band syndrome, posttraumatic knee stiffness, and gastrocnemius tendinopathy, ESWT should be cautiously applied for treating these KSTDs. Additional RCTs of the treatment effects of ESWT on KSTDs other than patellar tendinopathies are required to demonstrate the clinical efficacy of ESWT.

## Additional files

Additional file 1: **Table S1**. Search formulas for each database. (PDF 272 kb)

Additional file 2: **Table S2**. Summary of funding information and the declaration of conflict of interest for each included trial. (PDF 262 kb)

Additional file 3: **Figure S1**. Data and forest plot of clinical efficacy of extracorporeal shock wave therapy for the treatment success rate over the overall follow-up duration. (PDF 86 kb)

Additional file 4: **Figure S2**. Data and forest plot of clinical efficacy of extracorporeal shock wave therapy for the treatment success rate at each follow-up time point. (PDF 69 kb)

Additional file 5: **Figure S3**. Data and forest plot of clinical efficacy of extracorporeal shock wave therapy for pain reduction over the overall follow-up duration. (PDF 90 kb)

Additional file 6: **Figure S4**. Data and forest plot of clinical efficacy of extracorporeal shock wave therapy for pain reduction at each follow-up time point. (PDF 108 kb)

Additional file 7: **Figure S5**. Data and forest plot of clinical efficacy of extracorporeal shock wave therapy for patient-reported functional outcomes over the overall follow-up duration. (PDF 701 kb)

Additional file 8: **Figure S6**. Data and forest plot of clinical efficacy of extracorporeal shock wave therapy for patient-reported functional outcomes at each follow-up time point. (PDF 93 kb)

Additional file 9: **Figure S7**. Data and forest plot of clinical efficacy of extracorporeal shock wave therapy for performance-based functional outcomes over the overall follow-up duration. (PDF 60 kb)

Additional file 10: **Table S3**. Complications and adverse events in the included trials. (PDF 364 kb)

## Abbreviations

ACL: Anterior cruciate ligament
CI: Confidence interval
EFD: Energy flux density
ESWT: Extracorporeal shock wave therapy
FoSWT: Focused shock wave therapy
FU: Follow-up
KSTDs: Knee soft tissue disorders
LoE: Level of evidence
OR: Odds ratio
PEDro: Physiotherapy Evidence Database
RaSWT: Radial shock wave therapy
RCT: Randomized control trial
ROM: Range of motion
SD: Standard deviation
SMD: Standard mean difference
TSR: Treatment success rate
VAS: Visual analog scale
