# Supplementary material for: Efficacy of extracorporeal shock wave therapy for knee tendinopathies and other soft tissue disorders: a meta-analysis of randomized controlled trials
Source: BMC Musculoskelet Disord. 2018 Aug 2;19:278. doi: 10.1186/s12891-018-2204-6 (PMC6090995; doi:10.1186/s12891-018-2204-6)
Supplement: Supplementary file 1 — Table S1. Search formulas for each database. (PDF 272 kb) [file 12891_2018_2204_MOESM1_ESM.pdf]

Table S1. Database search formulas

Data base	Search terms for query
**Pubmed**	
#29	(#27) AND #28
#28	(Randomized controlled trial) OR Randomization
#27	(#25) OR #26
#26	(#1) AND #24
#25	(#1) AND #20
#24	((#21) OR #22) OR #23
#23	femur
#22	tibia
#21	patella
#20	((#17) OR #18) OR #19
#19	(knee joint) AND soft tissue disorder
#18	(knee joint) AND musculoskeletal disorder
#17	((((((((((((((#2) OR #3) OR #4) OR #5) OR #6) OR #7) OR #8) OR #10) OR #11) OR #12) OR #13) OR #14) OR #15) OR #16) OR #17
#16	Jumper's knee
#15	Osgood-Schlatter disease
#14	(fabella syndrome) OR popliteal cyamella
#13	(pes anserine tendinopathy) OR pes anserine bursitis
#12	iliotibial band friction syndrome
#11	apophysitis
#10	bursitis
#9	Tibial tuberosity osteochondritis
#8	tenosynovitis
#7	plica
#6	enthesopathy
#5	apicitis
#4	synovitis
#3	(((ligament injury/) OR ligament disorder/) OR ligament rupture/) OR desmitis
#2	((tendinitis) OR tendinopathy) OR peritendinopathy
#1	(((shockwave therapy) OR shock wave therapy) OR extracorporeal shockwave therapy) OR extracorporeal shock wave therapy

(continued)

Table S1. (continued)

Data base	Search terms for query
**Physiotherapy Evidence Database (PEDro)**	
	Body part: lower leg or knee
	Method: clinical trial
	Abstract & Title:
#1	extracorporeal shock wave therapy
#2	extracorporeal shockwave therapy
#3	shock wave therapy
#4	shockwave therapy
#5	shock wave
#6	shockwave
#7	Randomized controlled trial
#8	Randomization
#9	tendinopathy
#10	tendinitis
#11	ligament injury
#12	synovitis
#13	bursitis
#14	iliotibial band
#15	pes anserine/pes anserinus/pes
#16	popliteal cyamella
#17	jumper's knee
#18	soft tissue
#19	musculoskeletal
#20	patella
#21	patellar
#22	patello
#23	Patellofemoral
#24	pain syndrome

(continued)

Table S1. (continued)

Data base	Search terms for query
**Excerpta Medica dataBASE (EMBASE)**	
#1	extracorporeal shock wave therapy
#2	extracorporeal shockwave therapy
#3	shock wave therapy
#4	shockwave therapy
#5	shock wave
#6	shockwave
#7	#1 OR #2 OR #3 OR #4 OR #5 OR #6
#8	tendinitis'/exp OR tendinitis OR 'tendinopathy'/exp OR tendinopathy OR peritendinopathy
#9	('ligament'/exp OR ligament) AND (injury OR disorder)
#10	synovitis'/exp OR synovitis
#11	enthesopathy'/exp OR enthesopathy
#12	apicitis
#13	plica
#14	tenosynovitis'/exp OR tenosynovitis
#15	osteochondritis'/exp OR osteochondritis
#16	bursitis'/exp OR bursitis
#17	apophysitis' OR apophysitis
#18	iliotibial AND band
#19	pes AND (anserine OR anserinus)
#20	fabella AND syndrome OR (popliteal AND cyamella)
#21	'osgood schlatter'
#22	jumper AND knee
#23	running AND injury
#24	knee AND joint AND musculoskeletal AND disorder
#25	knee AND joint AND soft AND tissue AND disorder
#26	#8 OR #9 OR #10 OR #11 OR #12 OR #13 OR #14 OR #15 OR #16 OR #17 OR #18 OR #19 OR #20 OR #21 OR #22 OR #23 OR #24 OR #25 OR #26
#27	'patella'/exp OR patella OR 'patellar ligament'
#28	'tibia'/exp OR tibia
#29	'femur'/exp OR femur
#31	#27 OR #28 OR #29
#32	#7 AND #26
#33	#7 AND #31
#34	(#32 OR #33) AND [randomized controlled trial]/lim AND ([article]/lim OR [article in press]/lim) AND [humans]/lim

(continued)

Table S1. (continued)

Data base	Search terms for query
**Cochrane Library Database**	
#1	(shock wave) OR (shockwave)
#2	tendinitis or tendinopathy or peritendinopathy
#3	ligament and (injury or disorder)
#4	synovitis or enthesopathy or apicitis or plica or tenosynovitis or osteochondritis or bursitis or apophysitis
#5	(Tibial tuberosity osteochondritis) OR (pes anserine) OR (pes anserinus) OR (fabella syndrome) OR (popliteal cyamella) OR (Osgood-Schlatter disease) OR (Jumper's knee)
#6	(knee soft tissue disorder) or (knee musculoskeletal disorder)
#7	(patella) OR (patellar) OR (Patellofemoral)
#8	#2 OR #3 OR #4 OR #5 OR #6 OR #7
#9	(Randomized controlled trial) OR Randomization
#10	#1 AND #8 AND #9
**China knowledge resource integrated database**	
#1	(shock wave therapy) OR (shock wave therapy)
#2	(knee or patella) AND (tendinitis or tendinopathy or peritendinopathy)
#3	(knee or patella) AND ligament and (injury or disorder)
#4	synovitis or enthesopathy or apicitis or plica or tenosynovitis or osteochondritis or bursitis or apophysitis
#5	(Tibial tuberosity osteochondritis) OR (pes anserine) OR (pes anserinus) OR (fabella syndrome) OR (popliteal cyamella) OR (Osgood-Schlatter disease) OR (Jumper's knee)
#6	(knee soft tissue disorder) or (knee musculoskeletal disorder)
#7	#2 OR #3 OR #4 OR #5 OR #6 OR #7
#8	(Randomized controlled trial) OR Randomization
#9	#1 AND #7 AND #8
**Google Scholar**	
#1	allintitle: (extracorporeal shock wave therapy tendonitis OR tendinopathy) AND (Randomized controlled trial OR Randomization)
#2	allintitle: extracorporeal shockwave therapy tendonitis OR tendinopathy AND (Randomized controlled trial OR Randomization)
#3	allintitle: extracorporeal shock wave therapy ligament AND (Randomized controlled trial OR Randomization)
#4	allintitle: extracorporeal shock wave therapy Osgood OR Schlatter AND (Randomized controlled trial OR Randomization)
#5	allintitle: extracorporeal shockwave therapy patella OR patellar OR AND (Randomized controlled trial OR Randomization)patellofemoral
