# Supplementary material for: Efficacy of extracorporeal shock wave therapy for knee tendinopathies and other soft tissue disorders: a meta-analysis of randomized controlled trials
Source: BMC Musculoskelet Disord. 2018 Aug 2;19:278. doi: 10.1186/s12891-018-2204-6 (PMC6090995; doi:10.1186/s12891-018-2204-6)
Supplement: Supplementary file 2 — Table S2. Summary of funding information and the declaration of conflict of interest for each included trial. (PDF 262 kb) [file 12891_2018_2204_MOESM2_ESM.pdf]

Table S2 Summary of funding information and conflict of interest disclosure for each included trial

Study author (year) [reference]	Source name	Grant/Project Number	Conflict of interest
Chen (2014) [51]	National Science Council of Taiwan	NSC: 99-2314-B-037-011-MY3	None declared
Geng (2017) [90]	Science and Technology Bureau (Dazhou City, Sichuan Province, China)[§]	2015JY0170	No information
Guan (2015) [80]	No information	No information	No information
Huang (2017) [88]	Health and Family Planning Commission Scientific Research (Gvangjsih Bouxcuengh Swcigih)[§]	Z2014353	No information
Jiang (2016) [81]	No information	No information	No information
Khosrawi (2017) [48]	Isfahan University of Medical Sciences	393191	None declared
Liu (2016) [82]	Scientific and Technical Research Fund (Jiangxi Province Office of Education)[§]	GJJ 14679	No information
Taunton (2003) [83]	The Nike Research Foundation;<br>BC Sports Medicine Research Foundation;<br>Siemens AG; Sonorex	No information	Declared
Thijs (2017) [84]	No information	No information	None declared
Vetrano (2013) [85]	No information	No information	None declared
Wang (2014) [86]	National Science Council;<br>Chang Gung Research Fund	NSC96-2314-B-182A-143;<br>CMRPG8B1291	None declared

(Continued)

Table S2 Continued

Study author (year) [reference]	Source name	Grant/Project Number	Conflict of interest
Weckström (2016) [52]	No information	No information	No information
Wu (2009) [91]	No information	No information	No information
Wu (2016) [89]	No information	No information	No information
Yang (2007) [16]	No information	No information	No information
Zhang (2016) [92]	Health and Family Planning Major Research Program (Ningxia Hui Autonomous Region)[§]	2014-NW-036	No information
Zhang (2017) [17]	No information	No information	No information
Zhou (2015) [53]	No information	No information	No information
Zwerver (2011) [87]	Netherlands Organisation for Health Research and Development (ZonMW)	750.20.010.	Declared

[§]The name of the funding source was translated to English by using translation software (Ginger Software, Inc.).
