# Supplementary material for: Efficacy of extracorporeal shock wave therapy for knee tendinopathies and other soft tissue disorders: a meta-analysis of randomized controlled trials
Source: BMC Musculoskelet Disord. 2018 Aug 2;19:278. doi: 10.1186/s12891-018-2204-6 (PMC6090995; doi:10.1186/s12891-018-2204-6)
Supplement: Supplementary file 3 — Figure S1. Data and forest plot of clinical efficacy of extracorporeal shock wave therapy for the treatment success rate over the overall follow-up duration. (PDF 86 kb) [file 12891_2018_2204_MOESM3_ESM.pdf]

Study or Subgroup	ESWT Events	ESWT Total	Control Events	Control Total	Weight	Odds Ratio IV, Random, 95% CI
**1.13.1 Overall duration**						
Geng 2017	14	30	6	30	8.1%	3.50 [1.11, 11.02]
Huang 2017	26	34	12	31	8.5%	5.15 [1.76, 15.04]
Khosrawi 2017	20	20	19	20	2.6%	3.15 [0.12, 82.16]
Liu 2016	48	50	36	50	6.6%	9.33 [1.99, 43.68]
Taunton 2003	7	9	0	7	2.7%	45.00 [1.83, 1104.64]
Thijs 2017	21	21	26	29	3.0%	5.68 [0.28, 116.06]
Vetrano 2013	12	23	12	23	8.1%	1.00 [0.31, 3.18]
Wang 2014	26	26	26	27	2.6%	3.00 [0.12, 77.03]
Weckström 2016	6	11	7	10	5.7%	0.51 [0.09, 3.11]
Wu 2009	19	30	4	30	7.6%	11.23 [3.10, 40.71]
Wu 2016	29	31	22	31	6.3%	5.93 [1.16, 30.25]
Yang 2007	16	28	5	29	7.8%	6.40 [1.89, 21.68]
Zhang 2016	15	18	7	18	6.5%	7.86 [1.65, 37.40]
Zhang 2017	18	28	10	26	8.3%	2.88 [0.95, 8.70]
Zhou 2015	18	30	27	30	7.1%	0.17 [0.04, 0.67]
Zwerver 2011	20	31	10	31	8.5%	3.82 [1.33, 10.94]
**Subtotal (95% CI)**		**420**		**422**	**100.0%**	**3.36 [1.84, 6.12]**
Total events	315		229			

Heterogeneity: Tau² = 0.81; Chi² = 37.21, df = 15 (P = 0.001); I² = 60%

Test for overall effect: Z = 3.95 (P < 0.0001)

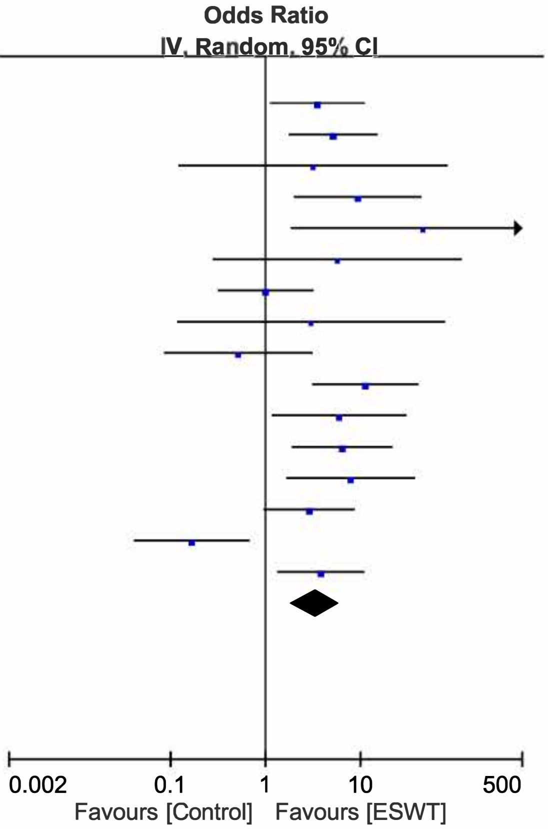

Figure S1. Forest plot of effects of extracorporeal shock wave therapy on treatment success rate at an overall duration. The horizontal line links the lower and upper limits of the 95% CI of this effect. The combined effects are plotted using black diamonds. ESWT = extracorporeal shock wave therapy; 95% CI = 95% confidence interval; Random = random-effects model; Std. = standard.
