# Supplementary material for: Efficacy of extracorporeal shock wave therapy for knee tendinopathies and other soft tissue disorders: a meta-analysis of randomized controlled trials
Source: BMC Musculoskelet Disord. 2018 Aug 2;19:278. doi: 10.1186/s12891-018-2204-6 (PMC6090995; doi:10.1186/s12891-018-2204-6)
Supplement: Supplementary file 4 — Figure S2. Data and forest plot of clinical efficacy of extracorporeal shock wave therapy for the treatment success rate at each follow-up time point. (PDF 69 kb) [file 12891_2018_2204_MOESM4_ESM.pdf]

	ESWT		Control			Odds Ratio
Study or Subgroup	Events	Total	Events	Total	Weight	IV, Random, 95% CI
**1.14.1 Long-term (>6 mon)**						
Huang 2017	26	34	12	31	25.0%	5.15 [1.76, 15.04]
Liu 2016	48	50	36	50	22.0%	9.33 [1.99, 43.68]
Vetrano 2013	15	23	21	23	21.0%	0.18 [0.03, 0.96]
Wang 2014	26	26	26	27	12.1%	3.00 [0.12, 77.03]
Weckström 2016	7	11	6	9	19.9%	0.88 [0.14, 5.58]
**Subtotal (95% CI)**		**144**		**140**	**100.0%**	**1.91 [0.43, 8.42]**
Total events	122		101			
Heterogeneity: Tau² = 2.00; Chi² = 15.20, df = 4 (P = 0.004); I² = 74%						
Test for overall effect: Z = 0.85 (P = 0.39)						
**1.14.2 Medium-term (>3 mon, ≤6 mon)**						
Thijs 2017	10	15	18	26	50.0%	0.89 [0.23, 3.46]
Vetrano 2013	13	23	19	23	50.0%	0.27 [0.07, 1.06]
**Subtotal (95% CI)**		**38**		**49**	**100.0%**	**0.49 [0.16, 1.56]**
Total events	23		37			
Heterogeneity: Tau² = 0.21; Chi² = 1.45, df = 1 (P = 0.23); I² = 31%						
Test for overall effect: Z = 1.20 (P = 0.23)						
**1.14.3 Shotr-term (>1 mon, ≤3 mon)**						
Khosrawi 2017	20	20	19	20	8.5%	3.15 [0.12, 82.16]
Thijs 2017	11	18	16	25	24.2%	0.88 [0.25, 3.09]
Vetrano 2013	12	23	12	23	25.3%	1.00 [0.31, 3.18]
Weckström 2016	6	11	4	9	18.3%	1.50 [0.26, 8.82]
Wu 2009	19	30	4	30	23.7%	11.23 [3.10, 40.71]
**Subtotal (95% CI)**		**102**		**107**	**100.0%**	**2.05 [0.69, 6.10]**
Total events	68		55			
Heterogeneity: Tau² = 0.88; Chi² = 10.08, df = 4 (P = 0.04); I² = 60%						
Test for overall effect: Z = 1.28 (P = 0.20)						
**1.14.4 Immediate (≤1 mon)**						
Geng 2017	14	30	6	30	11.6%	3.50 [1.11, 11.02]
Khosrawi 2017	19	20	15	20	6.7%	6.33 [0.67, 60.16]
Taunton 2003	7	9	0	7	4.2%	45.00 [1.83, 1104.64]
Thijs 2017	21	21	26	29	4.6%	5.68 [0.28, 116.06]
Weckström 2016	6	11	7	10	8.5%	0.51 [0.09, 3.11]
Wu 2016	29	31	22	31	9.2%	5.93 [1.16, 30.25]
Yang 2007	16	28	5	29	11.2%	6.40 [1.89, 21.68]
Zhang 2016	15	18	7	18	9.5%	7.86 [1.65, 37.40]
Zhang 2017	18	28	10	26	11.8%	2.88 [0.95, 8.70]
Zhou 2015	18	30	27	30	10.3%	0.17 [0.04, 0.67]
Zwerver 2011	20	31	10	31	12.1%	3.82 [1.33, 10.94]
**Subtotal (95% CI)**		**257**		**261**	**100.0%**	**3.09 [1.43, 6.69]**
Total events	183		135			
Heterogeneity: Tau² = 0.99; Chi² = 27.33, df = 10 (P = 0.002); I² = 63%						
Test for overall effect: Z = 2.86 (P = 0.004)						

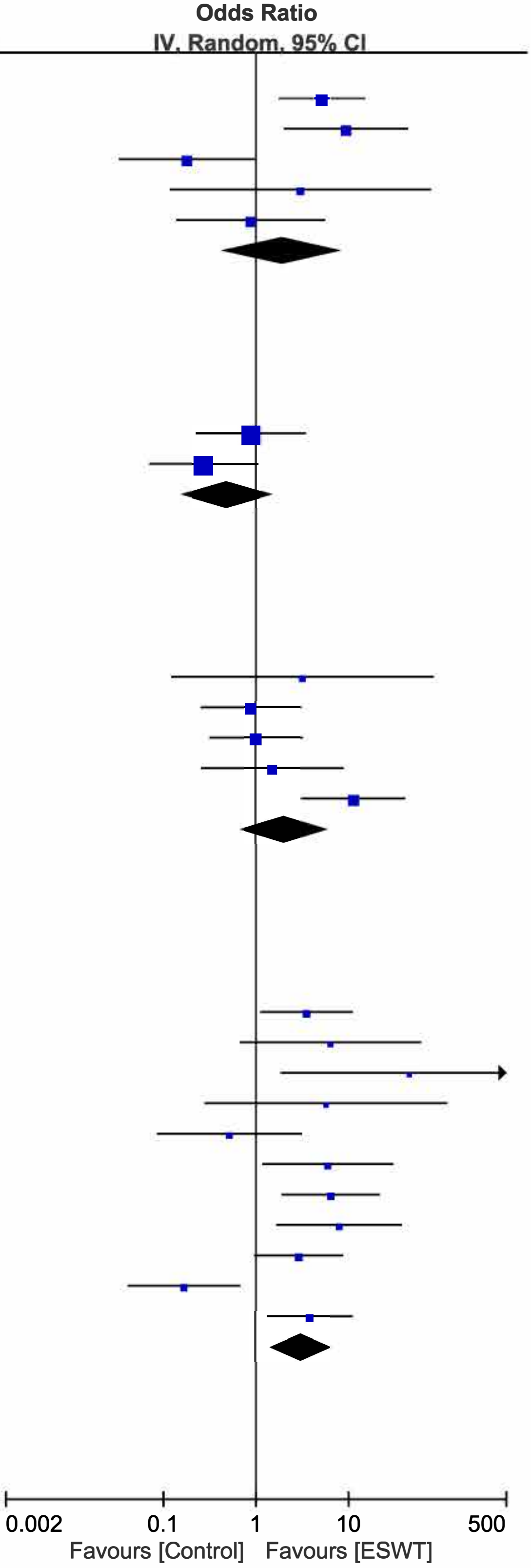

Figure S2. Forest plot of effects of extracorporeal shock wave therapy on treatment success rate at each follow up time point. The horizontal line links the lower and upper limits of the 95% CI of this effect. The combined effects are plotted using black diamonds. ESWT = extracorporeal shock wave therapy; 95% CI = 95% confidence interval; Random = random-effects model; Std. = standard.
