# Supplementary material for: Efficacy of extracorporeal shock wave therapy for knee tendinopathies and other soft tissue disorders: a meta-analysis of randomized controlled trials
Source: BMC Musculoskelet Disord. 2018 Aug 2;19:278. doi: 10.1186/s12891-018-2204-6 (PMC6090995; doi:10.1186/s12891-018-2204-6)
Supplement: Supplementary file 5 — Figure S3. Data and forest plot of clinical efficacy of extracorporeal shock wave therapy for pain reduction over the overall follow-up duration. (PDF 90 kb) [file 12891_2018_2204_MOESM5_ESM.pdf]

Study or Subgroup	ESWT Mean	ESWT SD	ESWT Total	Control Mean	Control SD	Control Total	Weight	Std. Mean Difference IV, Random, 95% CI
**2.13.1 Overall**								
Chen 2014, noninvasive active control	-36	2	30	9	3	30	2.3%	-17.42 [-20.69, -14.16]
Geng 2017	-51	9	30	-41	7	30	5.9%	-1.22 [-1.78, -0.67]
Guan 2015	-81	4	73	-70	8	73	6.0%	-1.73 [-2.11, -1.35]
Huang 2017	-44	6	34	-14	6	31	5.3%	-4.94 [-5.94, -3.94]
Jiang 2016	-36	9	40	-32	4	40	6.0%	-0.57 [-1.02, -0.12]
Khosrawi 2017	-47	33	20	-19	40	20	5.8%	-0.75 [-1.39, -0.11]
Liu 2016	-55	8	50	-36	8	50	5.9%	-2.36 [-2.87, -1.84]
Taunton 2003	-25	9	10	-3	4	10	4.7%	-3.03 [-4.39, -1.66]
Thijs 2017	-13	23	22	-14	25	30	5.9%	0.04 [-0.51, 0.59]
Vetrano 2013	-24	22	23	-34	19	23	5.8%	0.48 [-0.11, 1.07]
Weckström 2016	-69	61	11	-28	50	13	5.5%	-0.72 [-1.55, 0.12]
Wu 2009	-41	34	30	-15	33	30	5.9%	-0.77 [-1.29, -0.24]
Wu 2016	-58	6	31	-46	7	31	5.8%	-1.82 [-2.42, -1.22]
Yang 2007	-43	22	28	-16	21	29	5.9%	-1.24 [-1.81, -0.67]
Zhang 2016	-12	4	18	-8	4	18	5.7%	-0.98 [-1.67, -0.28]
Zhang 2017	-28	10	28	-16	12	26	5.8%	-1.07 [-1.65, -0.50]
Zhou 2015	-11	10	30	-19	11	30	5.9%	0.75 [0.23, 1.28]
Zwerver 2011	-8	8	31	-11	27	31	5.9%	0.15 [-0.35, 0.65]
**Subtotal (95% CI)**			**539**			**545**	**100.0%**	**-1.49 [-2.11, -0.87]**

Heterogeneity: Tau² = 1.61; Chi² = 320.69, df = 17 (P < 0.00001); I² = 95%

Test for overall effect: Z = 4.73 (P < 0.00001)

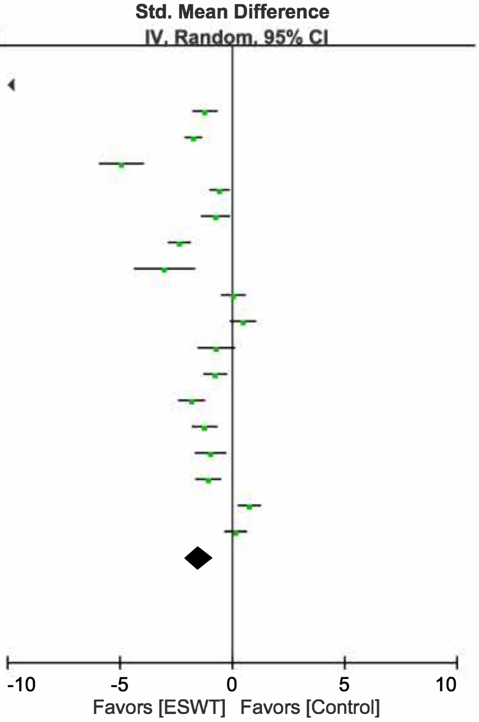

Figure S3. Forest plot of effects of extracorporeal shock wave therapy on pain reduction at an overall duration. The horizontal line links the lower and upper limits of the 95% CI of this effect. The combined effects are plotted using black diamonds. ESWT = extracorporeal shock wave therapy; 95% CI = 95% confidence interval; Random = random-effects model; Std. = standard.
