# Supplementary material for: Efficacy of extracorporeal shock wave therapy for knee tendinopathies and other soft tissue disorders: a meta-analysis of randomized controlled trials
Source: BMC Musculoskelet Disord. 2018 Aug 2;19:278. doi: 10.1186/s12891-018-2204-6 (PMC6090995; doi:10.1186/s12891-018-2204-6)
Supplement: Supplementary file 6 — Figure S4. Data and forest plot of clinical efficacy of extracorporeal shock wave therapy for pain reduction at each follow-up time point. (PDF 108 kb) [file 12891_2018_2204_MOESM6_ESM.pdf]

Study or Subgroup	ESWT Mean	ESWT SD	ESWT Total	Control Mean	Control SD	Control Total	Weight	Std. Mean Difference IV, Random, 95% CI
**2.14.1 Long-term follow up (>6 mon)**								
Huang 2017	-52	7	34	-39	7	31	33.3%	-1.83 [-2.42, -1.25]
Liu 2016	-55	8	50	-36	8	50	33.5%	-2.36 [-2.87, -1.84]
Vetrano 2013	-31	23	23	-51	32	23	33.2%	0.71 [0.11, 1.30]
**Subtotal (95% CI)**			**107**			**104**	**100.0%**	**-1.17 [-2.99, 0.66]**
Heterogeneity: Tau² = 2.52; Chi² = 62.71, df = 2 (P < 0.00001); I² = 97%								
Test for overall effect: Z = 1.25 (P = 0.21)								
**2.14.2 Medium-term follow up (>3 mon, ≤6 mon)**								
Chen 2014, noninvasive active control	-36	2	30	9	3	30	8.9%	-17.42 [-20.69, -14.16]
Guan 2015	-81	4	73	-70	8	73	18.4%	-1.73 [-2.11, -1.35]
Liu 2016	-46	8	50	-34	9	50	18.3%	-1.40 [-1.84, -0.96]
Thijs 2017	-17	23	22	-20	24	30	18.1%	0.13 [-0.43, 0.68]
Vetrano 2013	-24	22	23	-34	19	23	18.0%	0.48 [-0.11, 1.07]
Zwerver 2011	-8	8	31	-11	27	31	18.2%	0.15 [-0.35, 0.65]
**Subtotal (95% CI)**			**229**			**237**	**100.0%**	**-1.98 [-3.32, -0.64]**
Heterogeneity: Tau² = 2.50; Chi² = 172.49, df = 5 (P < 0.00001); I² = 97%								
Test for overall effect: Z = 2.90 (P = 0.004)								
**2.14.3 Shotr-term follow up (>1 mon, ≤3 mon)**								
Huang 2017	-44	6	34	-14	6	31	10.1%	-4.94 [-5.94, -3.94]
Khosrawi 2017	-47	33	20	-19	40	20	11.2%	-0.75 [-1.39, -0.11]
Liu 2016	-35	8	50	-20	9	50	11.7%	-1.75 [-2.21, -1.28]
Taunton 2003	-23	11	10	-9	12	10	10.2%	-1.16 [-2.13, -0.20]
Thijs 2017	-13	23	22	-14	25	30	11.5%	0.04 [-0.51, 0.59]
Vetrano 2013	-24	28	23	-34	26	23	11.4%	0.36 [-0.22, 0.95]
Weckström 2016	-69	61	11	-28	50	13	10.7%	-0.72 [-1.55, 0.12]
Wu 2009	-41	34	30	-15	33	30	11.6%	-0.77 [-1.29, -0.24]
Zwerver 2011	-7	5	31	-5	5	31	11.6%	-0.39 [-0.90, 0.11]
**Subtotal (95% CI)**			**231**			**238**	**100.0%**	**-1.07 [-1.84, -0.31]**
Heterogeneity: Tau² = 1.24; Chi² = 108.92, df = 8 (P < 0.00001); I² = 93%								
Test for overall effect: Z = 2.75 (P = 0.006)								
**2.14.4 Immediate follow up (1 mon)**								
Chen 2014, noninvasive active control	-33	16	30	-4	14	30	6.4%	-1.90 [-2.52, -1.29]
Geng 2017	-51	9	30	-41	7	30	6.5%	-1.22 [-1.78, -0.67]
Huang 2017	-34	7	34	-10	6	31	5.9%	-3.62 [-4.43, -2.82]
Jiang 2016	-36	9	40	-32	4	40	6.7%	-0.57 [-1.02, -0.12]
Khosrawi 2017	-23	6	20	-1	9	20	5.7%	-2.82 [-3.72, -1.92]
Liu 2016	-24	9	50	-14	10	50	6.7%	-1.04 [-1.46, -0.62]
Taunton 2003	-25	9	10	-3	4	10	4.6%	-3.03 [-4.39, -1.66]
Thijs 2017	-8	4	22	-16	37	30	6.5%	0.28 [-0.27, 0.83]
Weckström 2016	-47	57	11	-39	67	13	5.9%	-0.12 [-0.93, 0.68]
Wu 2009	-29	11	30	-15	12	30	6.5%	-1.20 [-1.75, -0.65]
Wu 2016	-58	6	31	-46	7	31	6.4%	-1.82 [-2.42, -1.22]
Yang 2007	-43	22	28	-16	21	29	6.5%	-1.24 [-1.81, -0.67]
Zhang 2016	-12	4	18	-8	4	18	6.2%	-0.98 [-1.67, -0.28]
Zhang 2017	-28	10	28	-16	12	26	6.4%	-1.07 [-1.65, -0.50]
Zhou 2015	-11	10	30	-19	11	30	6.5%	0.75 [0.23, 1.28]
Zwerver 2011	-8	4	31	-7	11	31	6.6%	-0.12 [-0.62, 0.38]
**Subtotal (95% CI)**			**443**			**449**	**100.0%**	**-1.18 [-1.67, -0.68]**
Heterogeneity: Tau² = 0.91; Chi² = 164.95, df = 15 (P < 0.00001); I² = 91%								
Test for overall effect: Z = 4.65 (P < 0.00001)								

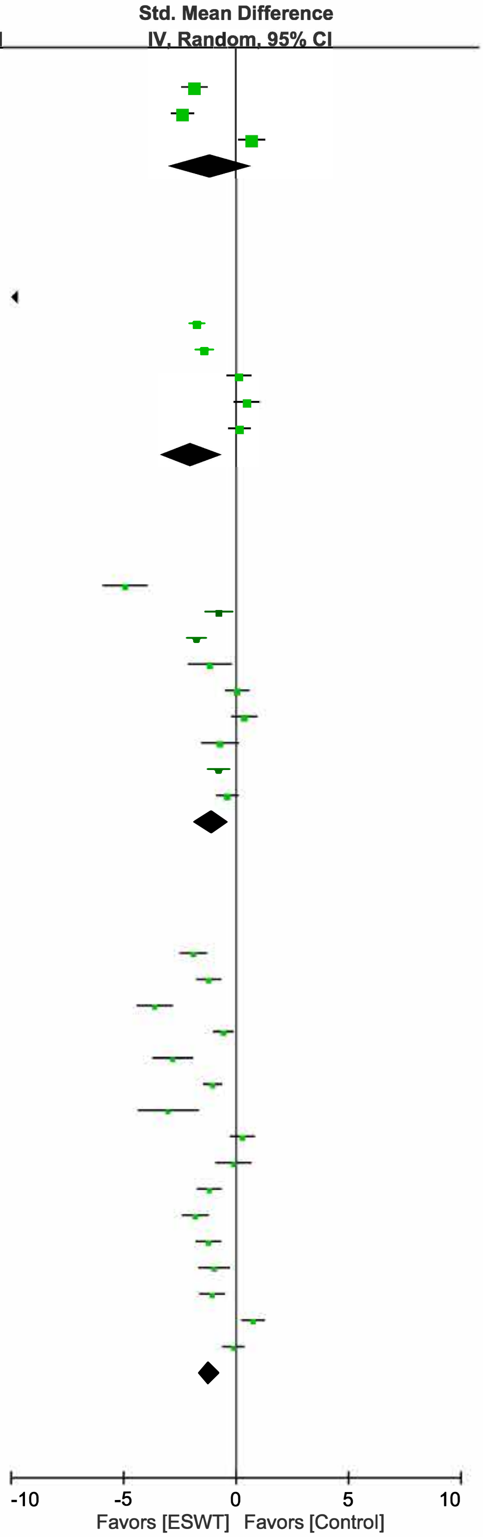

Figure S4. Forest plot of effects of extracorporeal shock wave therapy on pain reduction at each follow up time point. The horizontal line links the lower and upper limits of the 95% CI of this effect. The combined effects are plotted using black diamonds. ESWT = extracorporeal shock wave therapy; 95% CI = 95% confidence interval; Random = random-effects model; Std. = standard.
