# Supplementary material for: Efficacy of extracorporeal shock wave therapy for knee tendinopathies and other soft tissue disorders: a meta-analysis of randomized controlled trials
Source: BMC Musculoskelet Disord. 2018 Aug 2;19:278. doi: 10.1186/s12891-018-2204-6 (PMC6090995; doi:10.1186/s12891-018-2204-6)
Supplement: Supplementary file 7 — Figure S5. Data and forest plot of clinical efficacy of extracorporeal shock wave therapy for patient-reported functional outcomes over the overall follow-up duration. (PDF 701 kb) [file 12891_2018_2204_MOESM7_ESM.pdf]

Study or Subgroup	ESWT Mean	ESWT SD	ESWT Total	Control Mean	Control SD	Control Total	Weight	Std. Mean Difference IV, Random, 95% CI
**3.14.1 Overall**								
Chen 2014	5.6	0.4	30	0.4	0.7	30	6.4%	9.00 [7.26, 10.74]
Huang 2017	20.6	3.4	34	3.1	2.3	31	7.3%	5.90 [4.75, 7.06]
Jiang 2016	7.4	11.9	40	6.1	12.4	40	8.1%	0.11 [-0.33, 0.54]
Khosrawi 2017	20.5	13	20	11.7	14	20	7.9%	0.64 [0.00, 1.28]
Liu 2016	15.6	2.6	50	2.3	2.4	50	7.7%	5.27 [4.43, 6.12]
Taunton 2003	11.1	2.1	10	4.4	1.5	10	6.8%	3.52 [2.02, 5.02]
Thijs 2017	11.2	16.4	22	12.6	19.2	30	8.0%	-0.08 [-0.63, 0.47]
Vetrano 2013	15.2	19.5	23	20.9	15.5	23	8.0%	-0.32 [-0.90, 0.26]
Wang 2014	37.2	3	26	35.2	1.5	27	8.0%	0.84 [0.27, 1.40]
Wu 2009	6.2	1.8	30	3.3	2.3	30	8.0%	1.39 [0.82, 1.95]
Zhang 2016	46.3	15.5	18	36.1	13.1	18	7.9%	0.69 [0.02, 1.37]
Zhang 2017	27.6	8.6	28	19.1	7.1	26	8.0%	1.06 [0.49, 1.63]
Zwerver 2011	7.3	2.3	31	6.5	1.6	31	8.0%	0.40 [-0.10, 0.90]
**Subtotal (95% CI)**			**362**			**366**	**100.0%**	**2.03 [1.09, 2.96]**

Heterogeneity: Tau² = 2.76; Chi² = 319.77, df = 12 (P < 0.00001); I² = 96%

Test for overall effect: Z = 4.26 (P < 0.0001)

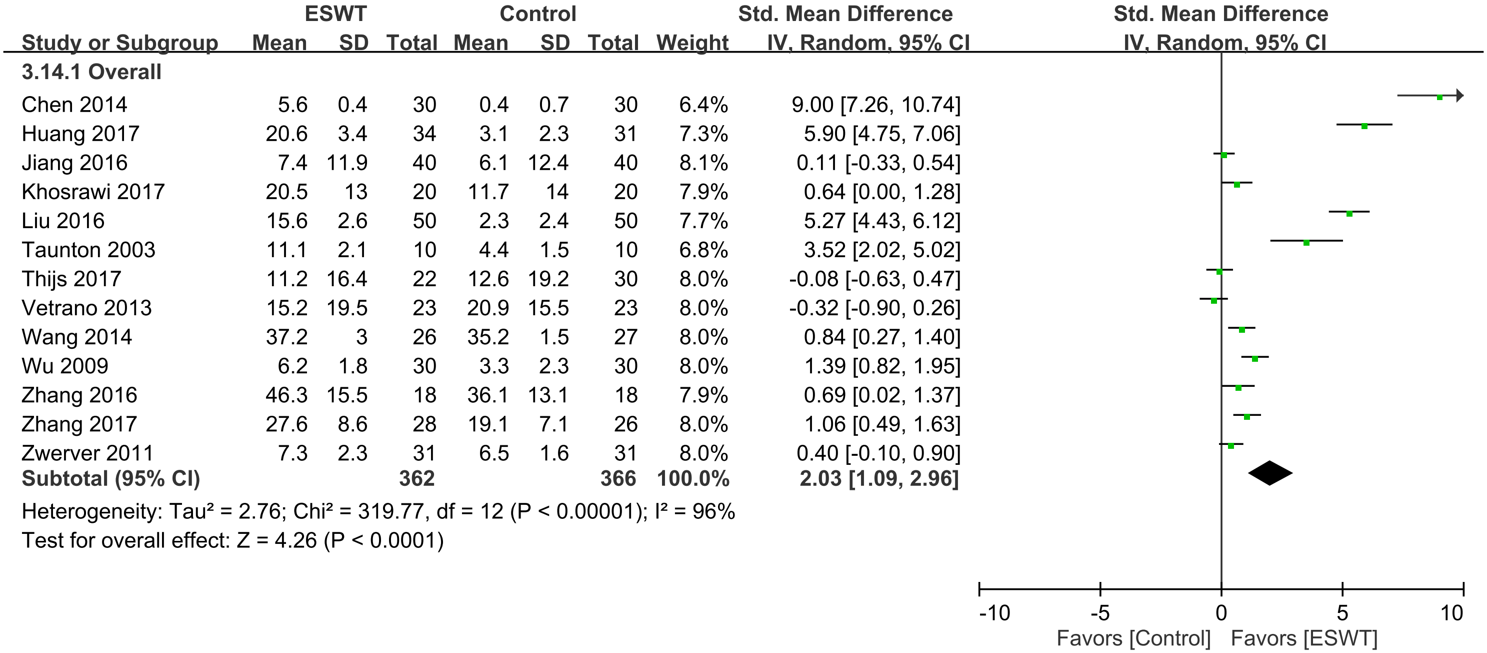

Figure S5. Forest plot of effects of extracorporeal shock wave therapy on patient-reported function outcome at an overall duration. The horizontal line links the lower and upper limits of the 95% CI of this effect. The combined effects are plotted using black diamonds. ESWT = extracorporeal shock wave therapy; 95% CI = 95% confidence interval; Random = random-effects model; Std. = standard.
