# Supplementary material for: Efficacy of extracorporeal shock wave therapy for knee tendinopathies and other soft tissue disorders: a meta-analysis of randomized controlled trials
Source: BMC Musculoskelet Disord. 2018 Aug 2;19:278. doi: 10.1186/s12891-018-2204-6 (PMC6090995; doi:10.1186/s12891-018-2204-6)
Supplement: Supplementary file 8 — Figure S6. Data and forest plot of clinical efficacy of extracorporeal shock wave therapy for patient-reported functional outcomes at each follow-up time point. (PDF 93 kb) [file 12891_2018_2204_MOESM8_ESM.pdf]

Study or Subgroup	ESWT Mean	ESWT SD	ESWT Total	Control Mean	Control SD	Control Total	Weight	Std. Mean Difference IV, Random, 95% CI
**3.15.1 Long-term follow up (>6 mon)**								
Huang 2017	25.3	2.4	34	15.6	2.3	31	24.8%	4.07 [3.20, 4.94]
Liu 2016	28	3.4	50	11.8	2.8	50	24.9%	5.16 [4.33, 5.99]
Vetrano 2013	21.5	19.9	23	36	12.7	23	25.1%	-0.85 [-1.46, -0.25]
Wang 2014	37.2	3	26	35.2	1.5	27	25.2%	0.84 [0.27, 1.40]
**Subtotal (95% CI)**			**133**			**131**	**100.0%**	**2.29 [-0.35, 4.92]**
Heterogeneity: Tau² = 7.09; Chi² = 171.82, df = 3 (P < 0.00001); I² = 98%								
Test for overall effect: Z = 1.70 (P = 0.09)								
**3.15.2 Medium-term follow up (>3 mon, ≤6 mon)**								
Chen 2014	5.6	0.4	30	0.4	0.7	30	18.1%	9.00 [7.26, 10.74]
Liu 2016	22.9	3.5	50	10.1	3.2	50	20.4%	3.79 [3.12, 4.45]
Thijs 2017	16.4	16.6	22	19.3	15.2	30	20.5%	-0.18 [-0.73, 0.37]
Vetrano 2013	17.6	22	23	31.4	28	23	20.5%	-0.54 [-1.13, 0.05]
Zwerver 2011	11.1	8.6	31	10.3	8.3	31	20.6%	0.09 [-0.40, 0.59]
**Subtotal (95% CI)**			**156**			**164**	**100.0%**	**2.28 [0.20, 4.35]**
Heterogeneity: Tau² = 5.37; Chi² = 202.71, df = 4 (P < 0.00001); I² = 98%								
Test for overall effect: Z = 2.15 (P = 0.03)								
**3.15.3 Shotr-term follow up (>1 mon, ≤3 mon)**								
Huang 2017	20.6	3.4	34	3.1	2.3	31	10.4%	5.90 [4.75, 7.06]
Khosrawi 2017	20.5	13	20	11.7	14	20	11.3%	0.64 [0.00, 1.28]
Liu 2016	22.2	3.2	50	6.8	3.3	50	11.1%	4.70 [3.93, 5.47]
Taunton 2003	6.9	1.8	10	3.3	1.5	10	10.4%	2.08 [0.95, 3.22]
Thijs 2017	11.2	16.4	22	12.6	19.2	30	11.4%	-0.08 [-0.63, 0.47]
Vetrano 2013	15.2	19.5	23	20.9	15.5	23	11.3%	-0.32 [-0.90, 0.26]
Wu 2009	9.4	6.1	30	6.5	4.3	30	11.4%	0.54 [0.03, 1.06]
Zhang 2016	46.3	15.5	18	36.1	13.1	18	11.2%	0.69 [0.02, 1.37]
Zwerver 2011	7.3	2.3	31	6.5	1.6	31	11.4%	0.40 [-0.10, 0.90]
**Subtotal (95% CI)**			**238**			**243**	**100.0%**	**1.56 [0.46, 2.67]**
Heterogeneity: Tau² = 2.72; Chi² = 205.09, df = 8 (P < 0.00001); I² = 96%								
Test for overall effect: Z = 2.77 (P = 0.006)								
**3.15.4 Immediate follow up (≤1 mon)**								
Chen 2014	4.2	0.6	30	0.6	0.3	30	8.2%	7.49 [6.01, 8.97]
Huang 2017	14.5	2.5	34	0.8	2.3	31	8.8%	5.62 [4.51, 6.73]
Jiang 2016	7.4	11.9	40	6.1	12.4	40	9.5%	0.11 [-0.33, 0.54]
Khosrawi 2017	15.5	13	20	6.8	12	20	9.3%	0.68 [0.04, 1.32]
Liu 2016	15.6	2.6	50	2.3	2.4	50	9.1%	5.27 [4.43, 6.12]
Taunton 2003	11.1	2.1	10	4.4	1.5	10	8.1%	3.52 [2.02, 5.02]
Thijs 2017	6.9	17.6	22	8.4	16.4	30	9.4%	-0.09 [-0.64, 0.46]
Wu 2009	6.2	1.8	30	3.3	2.3	30	9.4%	1.39 [0.82, 1.95]
Zhang 2016	19.5	1.3	18	18.8	1.2	18	9.3%	0.55 [-0.12, 1.21]
Zhang 2017	27.6	8.6	28	19.1	7.1	26	9.4%	1.06 [0.49, 1.63]
Zwerver 2011	7.4	13.6	31	3.8	13.6	31	9.5%	0.26 [-0.24, 0.76]
**Subtotal (95% CI)**			**313**			**316**	**100.0%**	**2.24 [1.16, 3.33]**
Heterogeneity: Tau² = 3.19; Chi² = 293.80, df = 10 (P < 0.00001); I² = 97%								
Test for overall effect: Z = 4.05 (P < 0.0001)								

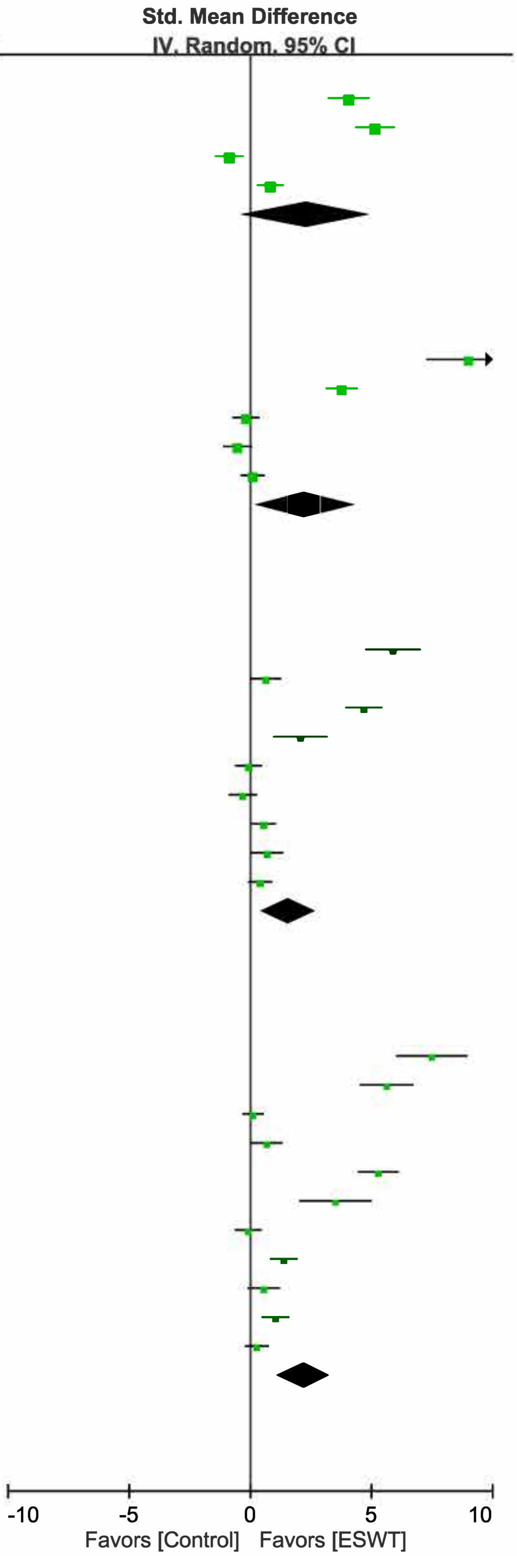

Figure S6. Forest plot of effects of extracorporeal shock wave therapy on patient-reported function outcome at each follow up time point. The horizontal line links the lower and upper limits of the 95% CI of this effect. The combined effects are plotted using black diamonds. ESWT = extracorporeal shock wave therapy; 95% CI = 95% confidence interval; Random = random-effects model; Std. = standard.
