# Supplementary material for: Efficacy of extracorporeal shock wave therapy for knee tendinopathies and other soft tissue disorders: a meta-analysis of randomized controlled trials
Source: BMC Musculoskelet Disord. 2018 Aug 2;19:278. doi: 10.1186/s12891-018-2204-6 (PMC6090995; doi:10.1186/s12891-018-2204-6)
Supplement: Supplementary file 9 — Figure S7. Data and forest plot of clinical efficacy of extracorporeal shock wave therapy for performance-based functional outcomes over the overall follow-up duration. (PDF 60 kb) [file 12891_2018_2204_MOESM9_ESM.pdf]

	ESWT			Control				Std. Mean Difference
Study or Subgroup	Mean	SD	Total	Mean	SD	Total	Weight	IV, Fixed, 95% CI
**4.2.1 FoSW, Range of motion**								
Chen 2014	26	8.6	30	1	9	30	48.0%	2.80 [2.08, 3.53]
Yang 2007	73.8	19	28	35.6	11	29	52.0%	2.44 [1.74, 3.13]
**Subtotal (95% CI)**			**58**			**59**	**100.0%**	**2.61 [2.11, 3.12]**
Heterogeneity: Chi² = 0.51, df = 1 (P = 0.48); I² = 0%								
Test for overall effect: Z = 10.20 (P < 0.00001)								
**4.2.2 RaSW, Range of motion**								
Zhang 2016	0.9	0.3	18	0.6	0.3	18	41.0%	0.98 [0.28, 1.67]
Zhang 2017	60	13.3	28	44	13.8	26	59.0%	1.16 [0.58, 1.74]
**Subtotal (95% CI)**			**46**			**44**	**100.0%**	**1.09 [0.64, 1.53]**
Heterogeneity: Chi² = 0.16, df = 1 (P = 0.69); I² = 0%								
Test for overall effect: Z = 4.78 (P < 0.00001)								
**4.2.3 Vertical jump test**								
Taunton 2003	1.5	0.8	10	0	0.5	10	100.0%	2.15 [1.00, 3.30]
**Subtotal (95% CI)**			**10**			**10**	**100.0%**	**2.15 [1.00, 3.30]**
Heterogeneity: Not applicable								
Test for overall effect: Z = 3.67 (P = 0.0002)								

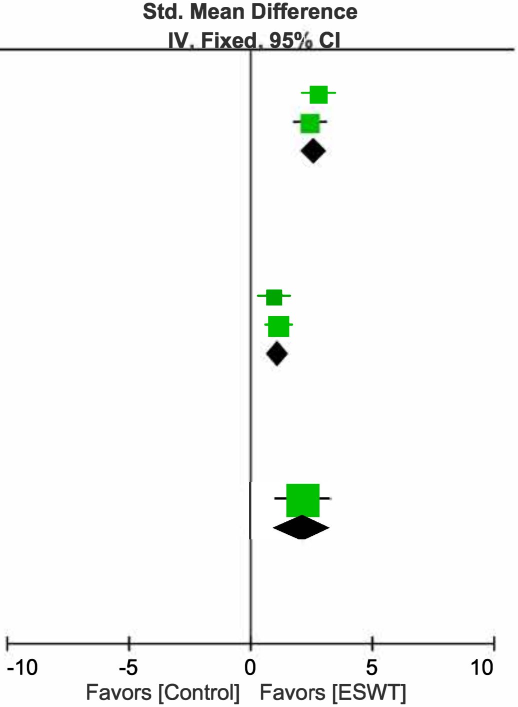

Figure S7. Forest plot of effects of extracorporeal shock wave therapy on performance-based function outcome at an overall duration. The horizontal line links the lower and upper limits of the 95% CI of this effect. The combined effects are plotted using black diamonds. ESWT = extracorporeal shock wave therapy; 95% CI = 95% confidence interval; Random = random-effects model; Std. = standard.
