# Supplementary material for: Efficacy of extracorporeal shock wave therapy for knee tendinopathies and other soft tissue disorders: a meta-analysis of randomized controlled trials
Source: BMC Musculoskelet Disord. 2018 Aug 2;19:278. doi: 10.1186/s12891-018-2204-6 (PMC6090995; doi:10.1186/s12891-018-2204-6)
Supplement: Supplementary file 10 — Table S3. Complications and adverse events in the included trials. (PDF 364 kb) [file 12891_2018_2204_MOESM10_ESM.pdf]

Table S3 Complications and adverse events in included trials

Shock-wave type	EG (number of events)							CG (number of events)						
**Study author (year) [reference]**	**Withdraw N (%)**	**Severe complications**	**Skin TR**	**Skin bruising**	**Pain DT**	**Pain AT**	**Others**	**Withdraw N (%)**	**Severe complications**	**Skin TR**	**Skin bruising**	**Pain DT**	**Pain AT**	**Others**
**Radial shock wave**														
Geng (2017) [90]	0 (0)	NA	NA	NA	NA	NA	NA	0 (0)	NA	NA	NA	NA	NA	NA
Huang (2017) [88]	0 (0)	N	N	N	N	N	N	0 (0)	N	N	N	N	N	N
Liu (2016) [82]	0 (0)	NA	NA	NA	NA	NA	NA	0 (0)	NA	NA	NA	NA	NA	NA
Weckström (2016) [52]	0 (0)	N	X (11)	N	N	N	N	4 (30.7)	N	N	X (<13)	N	N	N
Wu (2016) [89]	0 (0)	NA	NA	NA	NA	NA	NA	0 (0)	NA	NA	NA	NA	NA	NA
Guan (2015) [80]	NA	NA	NA	NA	NA	NA	NA	NA	NA	NA	NA	NA	NA	NA
Jiang (2016) [81]	0 (0)	NA	NA	NA	NA	NA	NA	0 (0)	NA	NA	NA	NA	NA	NA
Wu (2009) [91]	0 (0)	N	N	N	N	N	N	0 (0)	N	N	N	N	N	N
Zhang (2016) [92]	0 (0)	NA	NA	NA	NA	NA	NA	0 (0)	NA	NA	NA	NA	NA	NA
Zhang (2017) [17]	0 (0)	NA	NA	NA	NA	NA	NA	0 (0)	NA	NA	NA	NA	NA	NA
Zhou (2015) [53]	0 (0)	NA	NA	NA	NA	NA	NA	0 (0)	NA	NA	NA	NA	NA	NA
**Summary, No. of trials (No. of events)§**	0	0	1 (11)	0	0	0	0	0 (0)	0	0	1 (<13)	0	0	0
**Focused shock wave**														
Chen (2014) [51]	2 (6.7)	N	N	N	N	N	N	2 (10)	N	N	N	N	N	N
Thijs (2017) [84]	7 (31.8)	N	N	N	N	N	N	4 (13.3)	N	N	N	N	N	N
Vetrano (2013) [85]	1 (4.3)	N	X (≤23)	N	N	N	N	1 (4.3)	N	N	N	N	X (3)	N
Wang (2014) [86]	0 (0)	NA	NA	NA	NA	NA	NA	0 (0)	NA	NA	NA	NA	NA	NA
Zwerver (2011) [87]	1 (3.2)	N	N	N	N	N	N	3 (9.7)	N	N	N	N	N	N
Khosrawi (2017) [48]	NA	N	N	N	N	N	N	NA	N	N	N	N	N	N
Taunton (2003) [83]	NA	NA	NA	NA	NA	NA	NA	NA	NA	NA	NA	NA	NA	NA
Yang (2007) [16]	0 (0)	N	N	N	N	X (26)	N	6 (17.1)	N	N	N	N	X (29)	N
**Summary, No. of trials (No. of events)§**	4	0	1 (≤23)	0	0	1 (26)	0	5	0	0	0	0	2 (32)	0

§Total number of trials in which loss to follow-up and adverse events occurred.

The occurrence of adverse events is denoted as “X” along with the number of events. *N* = trial reporting no complication; NA = data on complications or adverse events were not available; TR = transient reddening; DT = during treatment; AT = after treatment; CG = control group; EG = experimental group; TED = total energy dose.
